# Gut microbial diversity and candidate keystone taxa in Indian tribes: Insights across lifestyle-ecological continuum and health associations

**DOI:** 10.1016/j.crmicr.2026.100650

**Published:** 2026-07-24

**Authors:** Sahid Afrid Mollick, Gin Khan Khual, Ankita Ghosh, Shiv Kumar Patel, Subhra Bhattacharyya, Chandra Sekhar Roy, Anwesh Maile, Hampapathalu Adimurthy Nagarajaram, Moajungla Longkumer, Mopada Nani Babu, Anup Raj Kundapur, Saloni Uniyal, Arna Chattterjee, Maitrayee Mitra, Mithun Sikdar, B.P. Urade, Venugopal N. Pulamaghatta

**Affiliations:** a DNA Laboratory, Anthropological Survey of India, Southern Regional Centre, Mysore, Karnataka, 5700026, India; b DNA Laboratory, Anthropological Survey of India, Head Office, Kolkata, West Bengal, 700091, India; c DBT-Centre for Microbial Informatics, Department of Systems and Computational Biology, School of Life Sciences, University of Hyderabad, Hyderabad, Telangana, 500046, India

**Keywords:** Gut microbiome, Tribe, Acculturation, Health association, Candidate keystone taxa, Lifestyle

## Abstract

Despite the critical role of the gut microbiome in host physiology and health, it remains poorly characterized in Indigenous populations undergoing rapid acculturation. This study presents high-resolution, whole-genome metagenomic profiling of gut microbiota from five Particularly Vulnerable Tribal Groups (PVTGs) of Southern India, Irula, Jenu Kuruba, Kurumba, Chenchu, and Konda Savara, spanning distinct ecological zones and cultural transitions. Using an ecology–lifestyle continuum framework, we investigated taxonomic and functional diversity with a focus on identifying computationally inferred candidate keystone taxa, defined by their association with variation in community ordination structure. A leave-one-taxon-out ordination framework identified 121 candidate keystone taxa, many of which were population-specific and have not been widely reported. Functional analyses revealed a conserved core of metabolic pathways, including glycolysis and folate biosynthesis, alongside group-specific enrichment in xenobiotic degradation, amino acid biosynthesis, mucin metabolism, and lipid processing, associated with differences in dietary and environmental exposures across populations. Large-scale disease-association mapping (n = 5,625) linked 50 candidate keystone taxa to 14 conditions, with 44 associated with health and 6 with disease. While *Fusicatenibacter saccharivorans* and *Alistipes shahii* were enriched in healthy states, *Ruminococcus gnavus, Bifidobacterium longum, Flavonifractor plautii*, and *Blautia wexlerae* were enriched in disease-associated profiles. Cross-cohort validation against an independent set of traditional metagenomes (n = 119) further showed that a subset of Indian tribal core candidate keystone taxa was consistently identified across geographically distinct populations, with partial conservation of community associations and health associations, indicating reproducible context-dependent microbial association patterns across traditional populations. Alpha diversity was highest in minimally acculturated groups, with higher degrees of acculturation associated with reduced microbial diversity and greater enrichment of disease-associated taxa. Overall, this study provides a context-aware framework for understanding gut microbiome dynamics in culturally transitioning populations, emphasizing the conservation of microbial heritage and informing population-specific microbiome-based interventions.

## Highlights

• An introduced analytical framework quantifies taxon–community associations to identify computationally inferred candidate keystone taxa.
• Application to five PVTGs identifies 121 candidate keystone taxa, most of which are population-specific, with a reproducible subset across traditional populations.
• Several candidate keystone taxa associated with lower community dispersion also show consistent associations with multiple disease conditions, highlighting priorities for future investigation.
• A conserved metabolic core coexists with population-specific functions, while acculturation is associated with reduced microbial diversity and increased disease-associated taxa.

## Graphical abstract

Graphical overview of gut microbiota composition across tribal populations and global disease datasets, identifying community-specific candidate keystone taxa, quantifying health and disease-associated microbes, and revealing microbiome shifts driven by lifestyle transitions.

**Figure:**
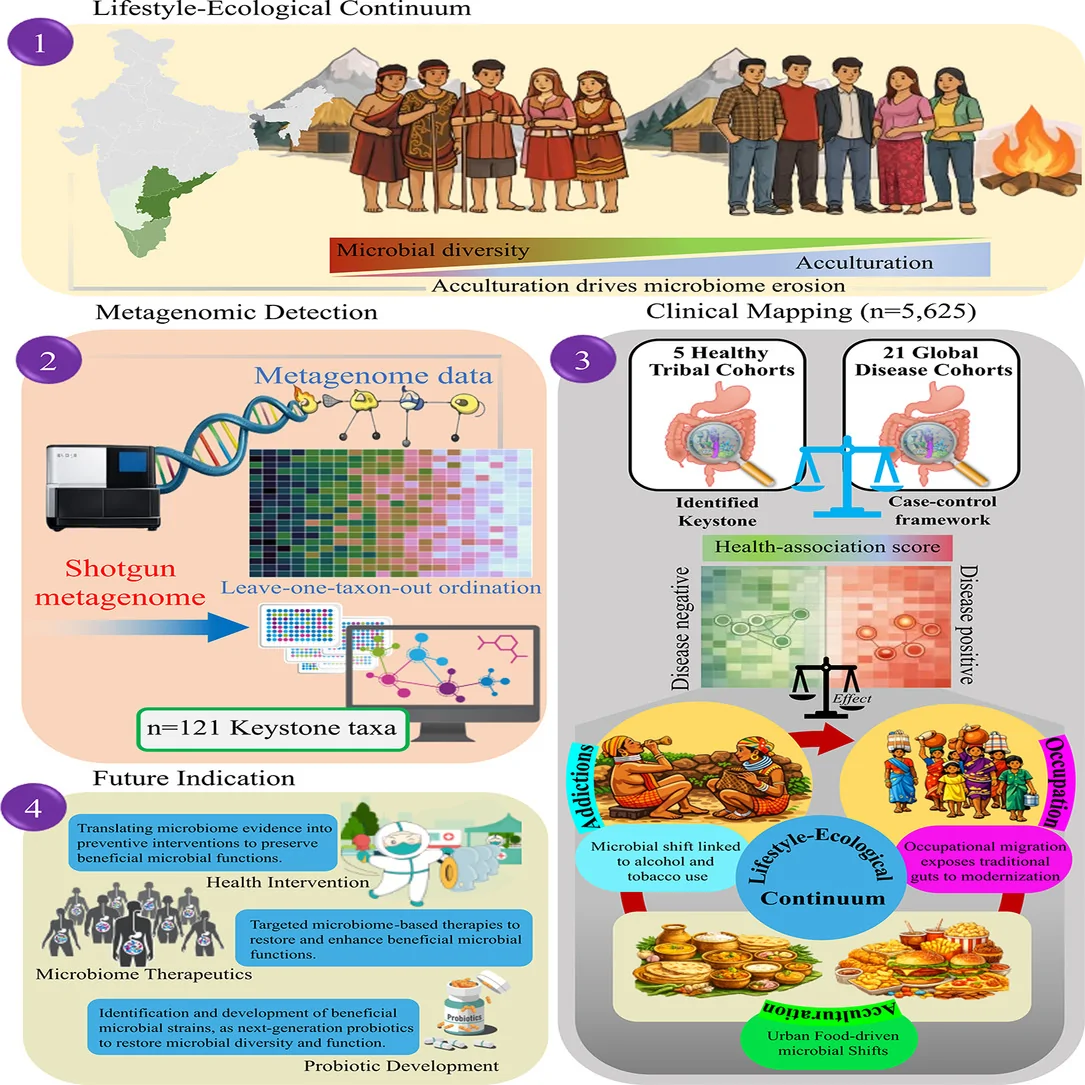

## Introduction

The human gut microbiome, comprising trillions of microbial symbionts, is essential for maintaining systemic physiological homeostasis (Paul et al., 2025). However, contrasting patterns underscore the plasticity of the human gut microbiome in response to lifestyle and environmental shifts, leading to marked alterations in gut microbial composition (Abjani et al., 2023). These transformations have raised significant concerns regarding their long-term impact on human health, particularly given the global rise in non-communicable diseases and autoimmune conditions (Collier and Venables 2017; United Nations, D,E,S,A., 2018; Newtonraj et al., 2017). In contrast, non-industrial societies, characterized by fiber-rich diets, physical exertion, and subsistence-based living, exhibit gut microbiota architectures markedly divergent from their industrialized counterpart. These configurations reflect not only dietary inputs but also sustained exposure to environmental microbiota, ethnobotanical practices, and co-evolved ecological symbioses (Carter et al., 2023; Rampelli et al., 2024; Moraïs et al., 2024; Mollick and Maji 2024). Such populations often retain distinct gut microbial compositions, offering valuable insights into host-microbiome interactions and their associations with health and disease (Mollick et al., 2025, Smits et al., 2017).

Indian tribal populations remain underrepresented in global microbiome research, despite their potential to harbour gut microbial communities that retain characteristics associated with traditional, less-industrialized lifestyles and limited exposure to antibiotics, processed foods, and urban pollutants (Oliveira et al., 2022; Yadav et al., 2023; Kim et al., 2024). Investigating gut microbiome variation across lifestyle–ecological gradients in these groups is key to understanding the microbial impacts of modernization (Vangay et al., 2018). Most Indian microbiome studies have relied on 16S rRNA sequencing, limiting taxonomic and functional insights, particularly among tribal populations (Bajaj et al., 2022; Chakraborty et al., 2024). Moreover, genetic studies indicate that tribal groups sharing geographic proximity tend to have similar genetic configuration over ethno-linguistic backgrounds (Mollick and Maji 2023) that may exhibit the shape of similar gut microbial abundance (Chakraborty et al., 2024).

Microbiome composition in Indian tribes is shaped not only by diet and environment but also by host genetics, ethno-linguistic diversity, and traditional cultural practices (Das et al., 2020). A multidisciplinary approach integrating microbiomics with ethnographic and ecological data is crucial to understand the impact of lifestyle transitions on health (Wang et al., 2023; Gurung et al., 2020). Acculturation-driven shifts in tribal microbiomes may reveal microbial signatures linked to metabolic and inflammatory disease risk. Comparing profiles across lifestyle gradients can help identify biomarkers of health vulnerability or resilience.

To address these gaps, we investigate the gut microbiome of five Particularly Vulnerable Tribal Groups (PVTGs) across South Indian states, spanning Telangana, Andhra Pradesh, Karnataka, and Tamil Nadu. This region, despite geographic continuity, varied cuisines, and ethno-linguistic lineages, making it an ideal setting to explore how ecological and lifestyle variations influence gut microbiome diversity among indigenous populations. This study focuses on identifying candidate keystone microbes that critically shape community stability to inform interventions that enhance gut resilience and prevent lifestyle-related diseases. However, identifying microbial candidate keystone taxa remains a major challenge in microbiome research because no universally accepted operational definition exists. Previous studies have inferred candidate keystone taxa using diverse criteria, including ecological network centrality, interaction strength, perturbation responses, and ecosystem function, each capturing different aspects of ecological importance and relying on distinct methodological assumptions (Faust and Raes 2012; Banerjee et al., 2018). Consequently, these approaches should be regarded as complementary rather than interchangeable (Garza et al., 2026). In the present study, we adopt an operational definition in which computationally inferred candidate keystone taxa are identified based on their disproportionate association with the multivariate organization of the gut microbial community following a leave-one-taxon-out ordination strategy. Rather than directly inferring ecological interactions or establishing causal ecosystem effects, this framework computationally prioritizes taxa whose removal is associated with marked changes in community structure, thereby generating biologically plausible candidates for subsequent validation using complementary network-based, experimental, or functional approaches.

Leveraging whole-genome shotgun metagenomic sequencing, this study aims to address three primary objectives: (i) to characterize the taxonomic composition, diversity, and functional potential of the gut microbiome across five PVTGs; (ii) to identify computationally inferred candidate keystone taxa using an ordination-based structural framework; and (iii) to evaluate disease-association of the identified candidate keystone signatures across populations representing a traditional-to-acculturated lifestyle continuum. Together, these complementary analyses provide an integrated structural and functional perspective on the gut microbiome of Indigenous populations undergoing lifestyle transition.

The observed differences in microbial composition and functional potential among the five tribal populations provided the biological basis for identifying microbial taxa that may play a disproportionately important role in shaping gut microbial communities. We hypothesized that, despite differences in community composition across populations, taxa that consistently show strong associations with overall community structure represent computationally inferred candidate keystone taxa. Furthermore, if these candidate keystone taxa reflect fundamental characteristics of healthy gut microbial communities rather than population-specific signatures, they should also exhibit reproducible associations with health status across independent disease cohorts and remain consistently identifiable in other traditional populations. Based on this hypothesis, the study was designed as a sequential analytical framework that first characterizes population-level microbial variation, then identifies candidate keystone taxa using an ordination-based approach, subsequently evaluates their health relevance across independent disease cohorts, and finally examines their robustness and reproducibility through cross-cohort validation.

## Methodology

### Ethical approval

Ethical approval for this study was obtained from the Institutional Human Ethics Committee (IHEC), Anthropological Survey of India, on 23 August 2023. The IHEC is registered with the National Ethics Committee Registry for Biomedical and Health Research (NECRBHR), Department of Health Research (DHR), Government of India (Registration No. EC/NEW/INST/2023/3910; registration dated 23 September 2023).

### Sampling

**Fig. 1:**
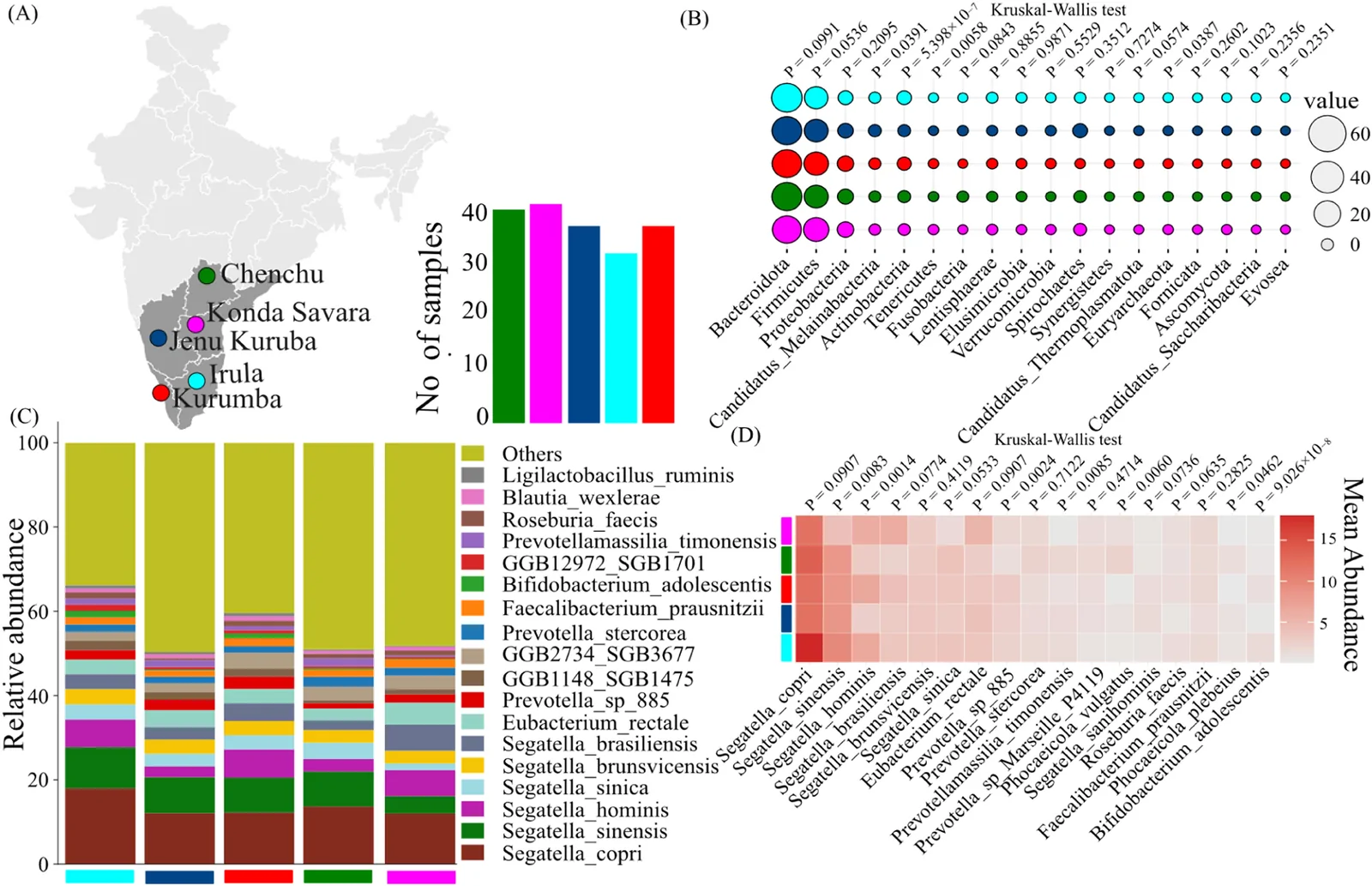
**Taxonomic structure and inter-population variability of gut microbiota across the studied tribal populations.** The spatial distribution of the sampled populations is illustrated in panel A, with each study site marked in population-specific colours and the respective state shaded in dark grey to contextualize geographic variation. Panel B presents the phylum-level microbial composition using a balloon plot, where the size of each circle reflects the relative abundance of the corresponding taxon, and significant inter-group differences are determined via the Kruskal–Wallis test (P < 0.05). Panel C illustrates the relative abundance of the top 20 most dominant microbial species across populations., highlighting both shared and unique population-specific taxonomic signatures. Finally, panel D heatmap showing microbial taxa contributing >1% to overall inter-population dissimilarity as identified by Similarity Percentage (SIMPER) analysis. Colours represent the log₁₀-transformed mean relative abundance of each taxon across populations. Statistical significance was evaluated using the Kruskal–Wallis test (P < 0.05).

A total of 182 fecal samples were obtained from individuals belonging to five distinct Particularly Vulnerable Tribal Groups (PVTGs) across southern India, using purposive sampling based on predefined inclusion and exclusion criteria. We inducted participants from the Irula (n = 36; Tamil Nadu), Kurumba (n = 36; Kerala), Jenu Kuruba (n = 31; Karnataka), Chenchu (n = 39; Telangana), and Konda Savara (n = 40; Andhra Pradesh) communities (Fig. 1A). Within each PVTG, samples were collected from two geographically distinct settings representing different stages along an acculturation continuum: (i) Less Acculturated (LA) areas, characterized by ecological remoteness and continued reliance on traditional subsistence practices with limited exposure to Western dietary and healthcare systems; and (ii) More Acculturated (MA) areas, characterized by greater integration into mainstream societal systems and increased access to modern diets, healthcare, and market-based resources. Acculturation was characterized using a structured qualitative ethnographic framework based on field observations and community interviews. Community classification was informed by multiple contextual indicators, including dietary patterns (traditional versus market-derived foods), reliance on self-procured versus purchased foods, food procurement and preparation practices, proximity to local markets, road connectivity, availability of subsidized foods through the public distribution system, and access to healthcare facilities. These indicators were considered collectively to characterize the degree of dietary and lifestyle transition and ecological exposure.

### Population recognition

Particularly Vulnerable Tribal Groups (PVTGs) constitute a highly marginalized subset of Scheduled Tribes, distinguished by characteristics such as pre-agricultural subsistence practices, low levels of literacy, persistent economic deprivation, geographic isolation, and declining or stagnant population growth (Anthropological Survey of India 2024). Following informed written consent, fecal samples were collected from apparently healthy male and female participants. Concurrently, structured socio-demographic data were obtained through standardized questionnaires (Supplementary Table S1).

### Study design

Personal information, including age, sex, anthropometric measurements, and preliminary biochemical test results, was collected from all participants. To ensure the inclusion of clinically healthy individuals, well-defined inclusion and exclusion criteria were applied. Exclusion criteria included individuals with a body mass index (BMI) below 18 kg/m² or above 25 kg/m²; those diagnosed with hypertension or hypotension; pregnant women, women planning to conceive, and lactating mothers. Individuals receiving long-term medication, including antibiotic use within the preceding 90 days, were also excluded. Additional exclusions applied to participants with any known comorbidities such as type 2 diabetes mellitus, retinopathy, neuropathy, nephropathy, cardiovascular diseases, a history of organ transplantation, or oral health disorders. The inclusion criteria were limited to apparently healthy male and female individuals aged 20–40 years, with no history of the aforementioned health conditions (Supplementary Table S2).

### Sample collection and isolation of DNA

Each participant was provided with detailed instructions and standardized training on the stool collection protocol. Stool samples were independently collected by the participants using 15 mL OMNIgene GUT collection tubes, specifically designed to preserve microbial DNA, ensuring sample integrity and standardized handling (DNA Genotek, OMR-200). Participants were instructed to collect their first-morning stool to ensure consistency and reduce diurnal variation. Immediately after collection, the samples were placed in portable iceboxes and transported under cold chain conditions (<10 °C) to the processing laboratory within 24 hours. Upon arrival, all samples were stored at 4 °C fridge, until further processing. Microbial genomic DNA was extracted from 250 µL aliquots of well-mixed stool homogenate using the QIAamp PowerFecal Pro DNA Kit (Qiagen, Cat. No. 51804), following the manufacturer's protocol optimized for efficient microbial lysis. The extracted DNA was eluted in 50 µL of molecular-grade nuclease-free water and stored at −20 °C until sequencing. DNA concentration and purity were measured spectrophotometrically using a NanoDrop 2000 (Thermo Fisher Scientific, USA), while DNA integrity and fragment size were assessed via 1% agarose gel electrophoresis. Samples exhibiting high-molecular-weight DNA and optimal A260/280 ratios (A260/280 ∼1.8–2.0) were selected for downstream analysis. Qualified DNA extracts were subjected to shotgun metagenomic sequencing on the Illumina NovaSeq 6000 platform.

### Sequencing strategy

Sequencing libraries were prepared using the Nextera DNA Flex Kit (Illumina, Inc.), following the manufacturer’s protocol. For each sample, a paired-end (PE) library with an average insert size of approximately 150 bp was constructed. Libraries were quantified and normalized using the Qubit assay, pooled, and sequenced on a single lane of the Illumina NovaSeq 6000 platform (NovaSeq Control Software v1.6.0/RTA v3.4.4) using a 2 × 150 bp configuration with the “NovaSeqXp” workflow in S1 mode flow cell. Base calling and demultiplexing were performed using bcl2fastq v2.20.0.422 from the CASAVA software suite. The quality score encoding followed the Sanger/phred33/Illumina 1.8+ format. Shotgun metagenomic sequencing was completed for all 182 samples, yielding an average of 24,473,941 reads per sample, with a maximum of 41,518,381 reads and a minimum of 12,753,984 reads (Supplementary Fig. 1).

### Bioinformatics analysis (raw data processing)

Raw paired-end shotgun metagenomic sequence data were initially subjected to quality assessment using FastQC v0.12.1 (Andrews 2010), and the resulting quality metrics across all samples were compiled using MultiQC (Ewels et al., 2016) to evaluate per-base sequence quality, nucleotide composition, and potential adapter contamination. Adapter trimming and removal of low-quality bases were performed using BBDuk (Bushnell 2014) under default parameters. To further improve data quality, Fastp (Chen et al., 2018) was employed to eliminate the duplicate reads. Only reads with a Phred quality score greater than 30, indicative of >99.9% base call accuracy, were retained for downstream analysis. To detect and eliminate host (human) DNA contamination, a two-step strategy was implemented. First, taxonomic classification of quality-filtered reads was performed using Kraken2 (Wood et al., 2019), which uses exact k-mer matching against a comprehensive reference database to identify human-derived sequences. This was followed by host read removal using the Hostile tool (Constantinides et al., 2023), which efficiently filters out sequences aligning to the human reference genome (GRCh38/hg38) while preserving microbial reads. This rigorous approach ensured high-confidence microbial read sets for all downstream analyses (Mollick 2025). Taxonomic profiling of host-depleted reads was carried out using MetaPhlAn v4.1 (Blanco-Míguez et al., 2023), which employs a clade-specific marker gene strategy for accurate and strain-resolved taxonomic classification. The analysis was performed using the mpa_vJun23_CHOCOPhlAnSGB_202403 database, which contains ∼5.1 million unique marker genes derived from over 1 million microbial genomes, including approximately 236,600 reference isolate genomes and 771,500 metagenome-assembled genomes (MAGs), covering a total of 26,970 species-level genome bins (SGBs). Following taxonomic annotation, functional profiling was performed using HUMAnN (HMP Unified Metabolic Analysis Network) (Franzosa et al., 2018). Functional gene alignment was conducted against the UniRef90 protein family database, followed by metabolic pathway reconstruction using the MetaCyc pathway database (Caspi et al., 2019). This integrated approach enabled robust annotation and quantification of gene families and metabolic pathways, thereby elucidating the functional potential of the gut microbiomes across the study populations.

### Statistical analysis

Statistical analyses were conducted in two sequential phases: an exploratory analysis to broadly characterize the microbial community structure, followed by a targeted analysis aimed at identifying key taxa with potential health relevance. Given the high dimensionality and inherent sparsity of microbiome count data, a two-step filtering strategy was employed to improve data quality and analytical robustness.

In the exploratory phase, a liberal filtering threshold was applied, retaining taxa with a minimum count of zero and presence in at least 20% of the samples. This approach preserved low-abundance and potentially rare taxa while eliminating highly sparse features, resulting in a dataset comprising 1,528 taxa. For the targeted analysis, a more stringent filtering criterion was applied, whereby only taxa with a minimum count of one and presence in at least 20% of the samples were retained. This refinement yielded a subset of 489 robust features suitable for downstream association analyses. Taxonomic abundances were subsequently normalized using Total Sum Scaling (TSS) to generate relative abundance profiles for downstream analyses. TSS was selected because it effectively accounts for differences in sequencing depth (library size) between samples and is a widely adopted normalization approach for relative abundance-based microbiome community analyses, including dissimilarity, constrained ordination, and differential abundance methods used in this study (Lin and Peddada, 2020). Although compositional approaches such as centered log-ratio (CLR) transformation are increasingly recommended for certain statistical applications, TSS was considered appropriate for the objectives and analytical framework employed here. (Justification in Supplementary Fig. 14).

Taxonomic composition was assessed at both the phylum and genus levels. To identify taxa contributing most to inter-group variation, SIMPER (Similarity Percentage) analysis was performed based on Bray–Curtis dissimilarity matrices. Taxa with an average dissimilarity contribution greater than 0.5% and a cumulative community contribution exceeding 1% were considered major contributors to observed microbial community differences.

Statistical comparisons of taxonomic abundance across populations were performed using the non-parametric Kruskal–Wallis test, based on median values, with statistical significance set at P < 0.05. Canonical Correspondence Analysis (CCA) was employed to visualize the relationships between microbial taxa and explanatory covariates, while distance-based Redundancy Analysis (db-RDA), using Bray–Curtis dissimilarity, was utilized to quantify the proportion of variance in microbial community composition attributable to environmental and demographic variables. The statistical significance of explanatory variables was evaluated using permutation-based tests, and variable importance was assessed using the coefficient of determination (R²) and corresponding P-values (< 0.05).

Within-sample (alpha) diversity was assessed by calculating observed richness. Pairwise differences between groups were evaluated using the Wilcoxon rank-sum test, with statistical significance determined after FDR (adjusted P values < 0.05). Between-sample (beta) diversity was assessed using Bray–Curtis dissimilarity. Differences in beta diversity across populations were tested using Permutational Multivariate Analysis of Variance (PERMANOVA). To identify differentially abundant taxa, DESeq2 was employed, which models count data using negative binomial generalized linear models. Differential abundance was determined based on log₂ fold change (log₂FC) estimates, with statistical significance defined by FDR adjusted P-values < 0.05 (Supplementary Fig. 9). Taxa meeting these criteria were considered significantly differentially abundant and were visualized in subsequent analyses.

To complement DESeq2-based findings, differential abundance of both microbial taxa and functional pathways was further assessed using Linear Discriminant Analysis Effect Size (LEfSe), which identifies taxonomic and functional biomarkers associated with population groups. Functional profiling was based on pathway abundance data generated by HUMAnN, using the UniRef90 gene family database and MetaCyc pathway annotations. Pathways contributing the most to inter-population variance were identified and subjected to LEfSe analysis to determine differentially abundant microbial functions. Throughout the analysis, population identity served as the primary grouping variable to capture microbial community patterns associated with varying degrees of traditional and acculturated lifestyles

In the second phase of analysis, we aimed to identify computationally inferred candidate keystone taxa, defined as taxa that are both *core-associated* and *influential* within microbial communities. To quantify the community-level impact of individual microbial taxa on overall compositional structure, we applied a modified version of the Empirical Presence–Absence Interrelation (EPI) framework (Amit and Bashan 2023), referred to as the Remove–Renormalize–Relate (RRR) approach (Goel et al., 2025), incorporating minor adaptations tailored to our dataset as a ‘leave-one-taxon-out ordination framework’. The leave-one-taxon-out framework integrates relative abundance information to quantify the structural influence of individual taxa on global microbiome structure and consists of three sequential steps. In the *leave* step, each target taxon was systematically excluded from the relative abundance matrix to assess its influence on community-wide structure. In the *taxon-out* step, the abundances of the remaining taxa were proportionally renormalized to preserve the compositional constraints of microbiome data, thereby isolating community-level variation independent of the focal taxon’s direct abundance contribution. In the *ordination* step, beta-diversity patterns of the renormalized profiles were summarized using Principal Coordinate Analysis (PCoA) based on Bray–Curtis dissimilarities, and the association between the excluded taxon’s original abundance and community structure was quantified by fitting its abundance vector onto the ordination space using the *envfit* function. Iterative application of this framework across all taxa yielded a continuous, abundance-weighted measure of taxon influence, enabling robust identification of taxa tightly coupled to overall microbiome configuration. This process was visualized using Principal Coordinates Analysis (PCoA) plots, exemplified by the contrasting influence of *Parabacteroides distasonis* (highly community-associated) and *Eubacterium tenue* (weakly community-associated) (Fig. 6).

Following this, all taxa were ranked according to three complementary metrics: the coefficient of determination (R²), the associated permutation-derived *P*-value from the *envfit* analysis, and prevalence within each population. To establish objective decision thresholds, prevalence and R² were evaluated using empirical sensitivity analyses. The prevalence sensitivity analysis identified a stable range between approximately 55% and 70%, from which a conservative threshold of ≥65% was selected to define consistently prevalent taxa. Similarly, evaluation of the empirical R² distribution identified the 65th percentile (R² ≥ 0.0199) as the threshold for strong association strength, providing an optimal balance between retaining candidate taxa and enriching the retained set for statistically supported associations (Justification in Supplementary Fig. 8). Accordingly, two distinct prevalence-defined categories were established. Core-associated taxa were defined as those satisfying R² ≥ 0.0199, permutation-derived P < 0.05, and prevalence ≥65%, whereas influential taxa were defined as those satisfying R² ≥ 0.0199, permutation-derived P < 0.05, and prevalence between 30% and 64%. Taxa satisfying either set of criteria were collectively designated as candidate keystone taxa, reflecting both their influence on microbial community structure and their population-level occurrence. The resulting association score was calculated as the number of cohorts in which each taxon satisfied these criteria and served as a quantitative measure of cross-population consistency. This framework was subsequently applied across all cohorts to identify taxa with high importance in microbial community organization. The formal expression of the identifying ‘candidate keystone taxa’ is:

$\begin{array}{c} C_{core}=\left\{t\in T:R^{2}\left(t\right)\geq 0.0199,P_{envfit}\left(t\right)<0.05,P_{prev}\left(t\right)\geq 65\%\right\},\\ C_{\inf}=\left\{t\in T:R^{2}\left(t\right)\geq 0.0199,P_{envfit}\left(t\right)<0.05,30\%\leq P_{prev}\left(t\right)<65\%\right\},\\ C=C_{core}\cup C_{\inf}. \end{array}$

The final analytical objective was to assess the association between the identified candidate keystone taxa and host health by evaluating their global disease relevance. To this end, we curated a comparative dataset comprising 5,625 whole-genome shotgun (WGS) metagenomic samples from 21 independent study cohorts spanning 18 countries. The dataset included 3,579 samples from individuals diagnosed with various diseases and 2,046 samples from healthy controls, all generated using whole-genome shotgun metagenomic sequencing (Supplementary Table S3). Because these comparative datasets were obtained from independent public studies, pre-analytical differences such as DNA extraction methods, library preparation protocols, sequencing platforms, and study-specific characteristics could not be standardized. To ensure analytical consistency, all publicly available raw sequencing datasets were reprocessed using the same bioinformatics workflow employed for the present study, including identical quality-control, taxonomic profiling, and downstream analytical procedures. Taxonomic abundance profiles were subsequently normalized to relative abundances prior to downstream analyses to improve comparability by reducing variation attributable to sequencing depth and library size. Cohorts were selected through a stepwise filtering process that ensured adequate representation of both disease and non-disease groups and minimized sampling bias toward any specific group.

The curated dataset encompassed 18 distinct disease conditions, which were further grouped into 14 broader disease categories (Supplementary Tables S4 and S5). To ensure equal statistical weighting across disease categories, regardless of the number of constituent cohorts corresponding to the same disease category, they were first aggregated. Within each disease category, differential abundance testing was performed between disease and non-disease groups using the Wilcoxon Rank-Sum test, focusing exclusively on the previously identified candidate keystone taxa (Fig. 7A).

Based on the outcome of these statistical comparisons, candidate keystone taxa were categorized into three groups according to the direction and statistical significance of differential abundance following false discovery rate (FDR) correction: SN (Significantly Negative), comprising taxa with significantly higher relative abundance in the non-disease group (FDR-adjusted q ≤ 0.15), indicating a potential health-associated role; SP (Significantly Positive), comprising taxa significantly enriched in the disease group (FDR-adjusted q ≤ 0.15), indicating a potential disease-associated role; and NS (Not Significant), including taxa showing no statistically significant difference between disease and non-disease groups (Fig. 7B).

This classification enabled exploratory characterization of candidate keystone taxa according to the consistency of their associations with health and disease across multiple disease categories. Because independent cohorts were pooled within disease categories, these analyses were intended for hypothesis generation rather than formal estimation of cross-cohort effect sizes.

To further quantify the consistency of these associations, a Health-Association Score (HS) was calculated for each candidate keystone taxon using the following equation:

$$\text{HS}=\frac{N_{SN}-N_{SP}}{N_{SN}+N_{SP}+N_{NS}}$$

where $N_{SN}$, $N_{SP}$, and $N_{NS}$ denote the number of disease categories in which a taxon was classified as Significantly Negative, Significantly Positive, or Not Significant, respectively. Consequently, HS values ranged from −1 to +1, with positive values indicating more frequent enrichment in healthy controls, negative values indicating more frequent enrichment in disease, and values near zero indicating inconsistent or neutral associations across disease categories.

To evaluate the robustness of Health-Association Score (HS) estimates, all possible combinations of 10 out of the 14 disease categories were exhaustively evaluated. This yielded 1,001 unique disease-category subsets (C(14,10) = 1,001), thereby eliminating stochastic variation associated with random resampling. For each subset, HS values were recalculated using the equation above, and the final HS for each taxon was obtained as the mean across all 1,001 subset-specific estimates. To quantify the stability of HS estimates across different disease-category compositions, the standard deviation (SD) of the subset-specific HS values was also calculated for each taxon (Supplementary Table S6). This exhaustive resampling strategy reduced dependence on any single disease category while providing a robust estimate of reproducible health and disease-associated microbial signatures. The resulting HS values were subsequently used to rank candidate keystone taxa according to their overall health association (Fig. 7C and Supplementary Table S6).

Integration of candidate keystone taxa profiles with their corresponding HS values enabled further stratification into functionally relevant groups reflecting their potential host-health associations. To further characterize the structural organization of candidate keystone taxa, microbial co-occurrence networks were constructed separately for each tribal population and for the combined dataset using normalized core taxon abundance profiles. Prior to network inference, abundance data were transformed using the centred log-ratio (CLR) transformation following the addition of a small pseudocount (1 × 10⁻⁶). Pairwise associations among taxa were estimated using Pearson correlation analysis with Benjamini–Hochberg-adjusted P-values. Only significant correlations with an absolute correlation coefficient of |*r*| ≥ 0.30 and an adjusted P < 0.05 were retained to capture moderate-to-strong microbial associations. The structural importance of individual taxa was quantified using degree, betweenness, and closeness centrality measures. Taxa were ranked according to these centrality metrics, and the top 15 taxa were designated as structurally central candidate keystone taxa for each population-specific and integrated network. To further characterize their interaction patterns, each top-ranked taxon was classified as exhibiting positive, negative, or dual interaction roles based on whether its significant network associations comprised exclusively positive correlations, exclusively negative correlations, or a combination of both. Subsequently, to investigate the distribution of disease-associated candidate keystone taxa across populations and host-related variables, differences in taxon abundance were evaluated using the Kruskal–Wallis test followed by pairwise Wilcoxon rank-sum tests. These analyses were restricted to taxa exhibiting significant community association (R², P < 0.05) and a prevalence of at least 30% across the tribal populations. Finally, the robustness and generalizability of the identified candidate keystone taxa were evaluated using an independent validation cohort comprising 119 individuals from four traditional populations (Supplementary Table S7).

Candidate keystone taxa identified in the discovery cohort (n = 121) were mapped to the validation cohort using the same taxonomic resolution and abundance preprocessing workflow. Taxa were considered validated if they satisfied the predefined criteria of significant community association (P < 0.05), prevalence ≥ 30%, and the corresponding R² threshold in the validation cohort (Fig. 6A).

Subsequently, the stability of candidate keystone taxa identified in both the discovery and validation cohorts was assessed using a cross-population framework. Because the validation dataset comprised four independent populations, all four possible combinations of three populations (C(4,3) = 4) were exhaustively evaluated rather than randomly sampled. For each subset, taxon-wise correlations between abundance and community dispersion were calculated using both Bray–Curtis and Aitchison distances. Population-specific effects were integrated using random-effects meta-analysis, with significance controlled by FDR-adjusted q-values. A composite stability score was calculated for each subset as the mean of the Bray–Curtis- and Aitchison-based stability estimates. The final stability score for each taxon was obtained as the mean across all four subset-specific estimates, and the corresponding standard deviation was calculated to quantify the robustness of stability estimates across population subsets (Supplementary Table S11). To further evaluate cross-cohort reproducibility, taxa were ranked according to their mean stability scores in the discovery and validation cohorts, and concordance of stability rankings was assessed using Spearman's rank correlation. Absolute rank differences were subsequently calculated for each taxon to identify taxa exhibiting concordant stability rankings (small rank differences) and discordant stability rankings (large rank differences) between cohorts (Supplementary Fig. 15 and Supplementary Table S11). Taxa exhibiting consistently high stability across all evaluated population subsets and high core score were considered robust candidate keystone members and were classified into four categories: (i) high core & high stability, (ii) high core only, (iii) high stability only, and (iv) low core & low stability. Candidate keystone reproducibility was defined as taxa meeting these criteria and consistently detected across multiple populations within the validation cohort (Fig. 7D and Supplementary Fig. 15).

All statistical analyses were performed using R version 4.5.0 (R Core Team 2025), and detailed scripts and resources are provided in the Supplementary Resource File.

## Result and analysis

### Taxonomic composition across the populations

We first characterized the overall taxonomic composition of the gut microbiota across the study populations. Taxonomic profiling was performed using clade-specific marker gene classification through MetaPhlAn, employing an updated database. Taxonomic relative abundances were normalized using Total Sum Scaling (TSS) (Justification in Methods Section). Across all samples, we identified a total of 18 phyla comprising 13 bacterial, 3 eukaryotic, and 2 archaeal lineages. The microbial richness varied across populations, with Konda Savara exhibiting the highest phylum-level richness (n = 17), followed by Chenchu (n = 16), Irula (n = 15), Jenu Kuruba (n = 14), and Kurumba (n = 13) (Supplementary Figures 2 and 3). The primary variation in microbial composition was driven by a trade-off in the relative abundances of *Bacteroidota* and *Firmicutes*, consistently observed across all study sites. *Bacteroidota* was the most dominant phylum, accounting for over 55% of the total relative abundance, followed by *Firmicutes* (>30%), *Proteobacteria* (>3%), and *Actinobacteria* (∼1%), while the remaining phyla contributed marginally. Significant inter-population differences in phylum-level abundance were detected for *Actinobacteria* (P = 5.398 × 10⁻⁷), *Tenericutes* (P= 0.0058), *Euryarchaeota* (P = 0.038), and *Candidatus Melainabacteria* (P = 0.039). For instance, *Actinobacteria* abundance was 2.11-fold higher in Irula, while *Spirochaetes* were 4.55-fold more abundant in Jenu Kuruba compared to other populations. Notably, *Candidatus Saccharibacteria* and *Evosea* exhibited specific enrichment in Chenchu and Konda Savara, respectively, while *Fornicata* showed specific enrichment in Irula and Konda Savara. Conversely, *Synergistetes* was absent in the Kurumba population (Supplementary Figs. 2, 3, and Fig. 1B).

At the species level, a total of 489 distinct microbial taxa were identified across the study populations, exhibiting considerable variation in both richness and compositional structure (Methods Section). Among these, *Segatella* species emerged as the most dominant genus, collectively accounting for more than 30% of the total species-level relative abundance. Other prominent species included *Eubacterium rectale* (4.14%), *Prevotella sp. 885* (2.37%), and *Faecalibacterium prausnitzii* (1.92%), which were consistently observed across the indigenous groups studied (Fig. 1C).

To identify the principal microbial contributors to compositional divergence among populations, we performed a Similarity Percentage (SIMPER) analysis based on Bray–Curtis dissimilarity. The analysis revealed that 17 species collectively explained 53.32% of the total between-population dissimilarity, indicating that a relatively small subset of taxa accounted for more than half of the observed compositional variation. Among these, *Segatella copri* was the single largest contributor, explaining 9.66% of the total dissimilarity, followed by *Segatella sinensis* (5.87%), *Segatella hominis* (4.97%), *Segatella brasiliensis* (3.72%), *Segatella brunsvicensis* (3.09%), *Segatella sinica* (2.91%), and *Eubacterium rectale* (2.84%). Together, these seven taxa accounted for approximately 33.1% of the total inter-population dissimilarity, highlighting the dominant role of the genus *Segatella* in driving microbial differentiation across the studied tribal populations.

Subsequently, statistical testing demonstrated that seven of these high-contributing taxa also exhibited significant differences in relative abundance among populations, including *Segatella sinensis* (P = 0.0083), *Segatella hominis* (P = 0.0014), *Prevotella* sp. 885 (P = 0.0085), *Phocaeicola vulgatus* (P = 0.0060), *Phocaeicola plebeius* (P = 0.0462), and *Bifidobacterium adolescentis* (P = 9.03 × 10⁻⁸) (Fig. 1D). The concordance between high SIMPER contribution and statistical significance indicates that these taxa not only contribute substantially to overall community dissimilarity but also represent robust population-specific microbial signatures.

To identify the factors exerting the strongest influence on gut microbial compositional variation, we conducted distance-based redundancy analysis (dbRDA) together with Canonical Correspondence Analysis (CCA), incorporating a range of population and lifestyle-related covariates. The dbRDA revealed that population identity explained the largest proportion of variation in microbiome composition (3.07% variance explained; adjusted R² = 0.0307, P = 0.001), followed by sex (0.76%; adjusted R² = 0.0076, P = 0.003) and degree of acculturation (0.66%; adjusted R² = 0.0066, P = 0.005).

These findings were further supported by the CCA ordination, which demonstrated population-level structuring of microbial communities together with associations with lifestyle-related variables (e.g., acculturation and habits and practices) (Fig. 5 and Supplementary Figures 5 and 6).

Collectively, these multivariate analyses identified population identity as the primary determinant of gut microbial compositional variation. Accordingly, all subsequent taxonomic and functional analyses were organized and interpreted on a population-specific basis to characterize the microbial features associated with each population.

**Fig. 2:**
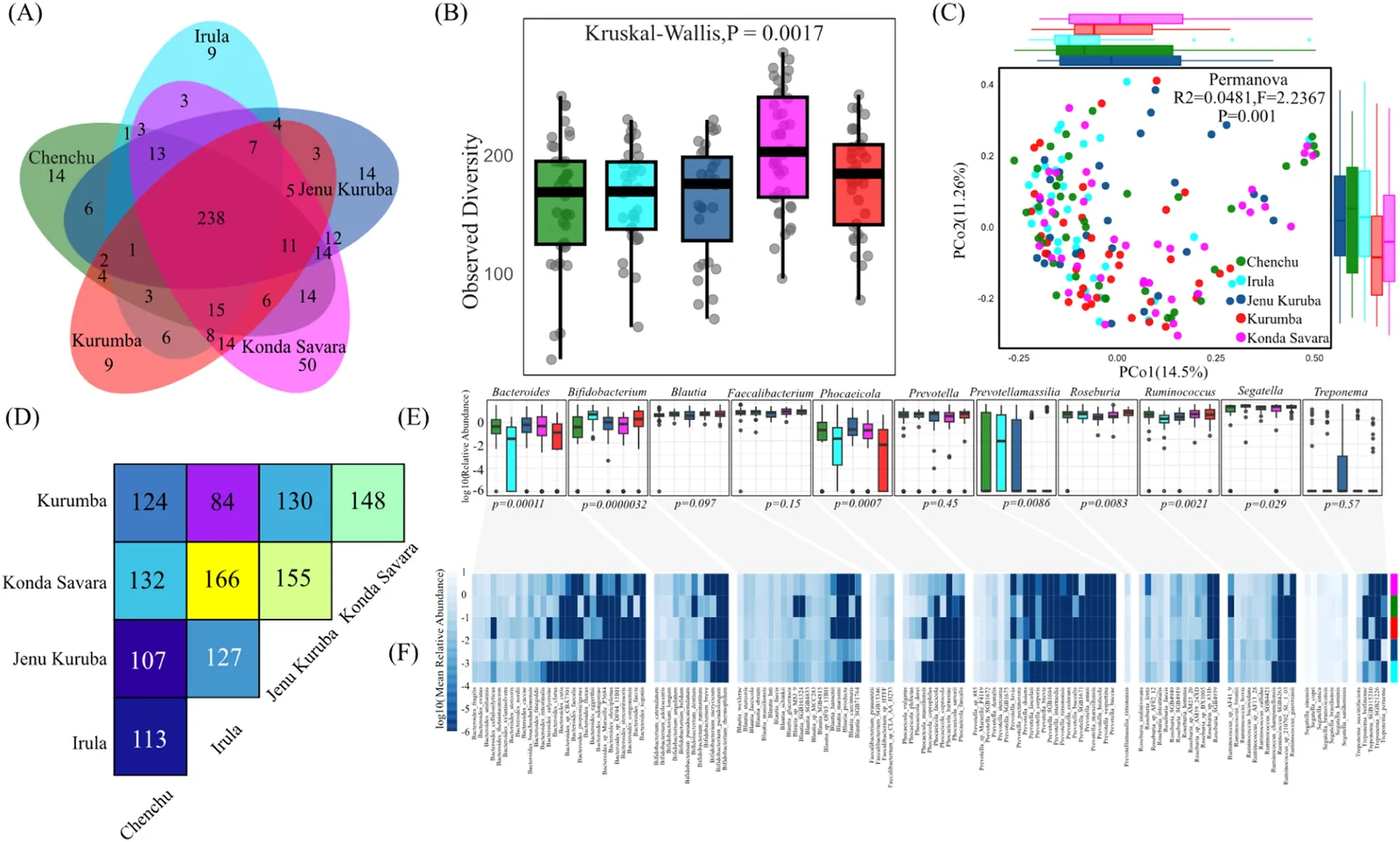
**Inter-site microbial diversity and compositional differentiation across studied tribal population cohorts.** Panel A illustrates the shared and population-specific taxonomic components among populations using a Venn diagram, highlighting patterns of overlap and population-specific species-level microbial signatures. Microbial alpha diversity, measured by observed richness, is compared across populations in panel B, revealing significant inter-group differences (Kruskal–Wallis test, P = 0.0017), suggestive of heterogeneous ecological exposures or lifestyle-associated factors. Beta diversity patterns are visualized in panel C through Principal Coordinate Analysis (PCoA) based on Bray–Curtis dissimilarity of species-level profiles, with samples color-coded by study site and distributional variation depicted along the first two principal axes. Panel D displays a heatmap of the number of species exhibiting high generalized fold changes between populations, with clustering based on the extent of taxonomic differentiation. In panel E, genera contributing more than 1% to overall population-level differences and cumulatively accounting for over 55% of the gut microbial community are shown based on log₁₀-transformed relative abundances, revealing key taxa driving compositional divergence. Panel F further resolves this variation by showing the log₁₀ mean relative abundance of all constituent species within these dominant genera across each population.

To further investigate inter-site differences in gut microbial composition, we assessed the distribution, diversity, and abundance of microbial taxa across the studied tribal populations. Notably, the Konda Savara population harboured the highest number of population-specific taxa (n = 50), while a core set of 238 taxa was detected across all populations. The greatest degree of taxonomic overlap was observed among the Irula, Chenchu, Kurumba, and Konda Savara populations (15 shared taxa), whereas the Jenu Kuruba population exhibited comparatively limited microbial overlap with the other groups. Interestingly, the Kurumba and Konda Savara populations shared 14 taxa, indicating a potential ecological or cultural linkage (Fig. 2A).

Observed species richness differed significantly among the five tribal populations (Kruskal-Wallis χ² = 17.34, df = 4, P = 0.0017; Fig. 2B), with a moderate effect size (0.075). Post hoc Dunn's tests with Benjamini-Hochberg correction showed that the Konda Savara population exhibited significantly higher richness than Chenchu (adjusted P = 0.005), Irula (adjusted P = 0.004), and Jenu Kuruba (adjusted P = 0.006), whereas no other pairwise comparisons remained significant. Pairwise effect sizes were of moderate magnitude (0.42–0.45) for these significant contrasts. Within-population comparisons between less acculturated (LA) and more acculturated (MA) individuals revealed significant differences in observed microbial richness for the Chenchu and Jenu Kuruba populations. Chenchu individuals from LA environments exhibited higher richness than those from MA environments (median = 228 vs. 173.5 taxa; raw P = 0.0189, FDR-adjusted P = 0.0472; effect size r = 0.40, moderate). Similarly, Jenu Kuruba individuals from MA environments showed significantly higher richness than those from LA environments (median = 229.5 vs. 193 taxa; raw P = 2.83 × 10⁻⁴, FDR-adjusted P = 0.0014; effect size 0.34, moderate). In contrast, Irula, Konda Savara, and Kurumba showed no significant differences following FDR correction (FDR-adjusted P > 0.05), with all comparisons exhibiting small effect sizes (0.16–0.24). (Supplementary Fig. 4). This site-specific diversity pattern was mirrored in beta diversity analysis based on principal coordinate analysis (PCoA), where population structure accounted for a significant proportion of variation (PERMANOVA p = 0.001, R² = 0.048). The first two principal coordinates explained a combined 25.7% of the total variance (PCoA1 = 14.5%; PCoA2 = 11.26%) (Fig. 2C).

Taxonomic analysis revealed site-specific differences in both abundance and prevalence of microbial taxa (Supplementary Table S8). Comparative analysis across populations with similar subsistence strategies identified 84 differentially abundant species between Irula and Kurumba, and 107 species between Chenchu and Jenu Kuruba. In contrast, greater differences were observed between populations with distinct ecological and lifestyle characteristics. For instance, 155 species differed between Jenu Kuruba and Konda Savara, and 166 species between Konda Savara and Irula (Fig. 2D and Supplementary Fig. 9). Among the taxa with mean relative abundance differences exceeding 1% based on Bray–Curtis dissimilarity, several genera exhibited statistically significant intergroup variation, including *Bacteroides* (P= 0.00011), *Bifidobacterium* (P = 3.2 × 10⁻⁶), *Phocaeicola* (P = 0.0007), *Prevotellamassilia* (P = 0.0086), *Roseburia* (P = 0.0083), *Ruminococcus* (P = 0.0021), and *Segatella* (P = 0.029). Other genera, such as *Blautia, Faecalibacterium, Prevotella*, and *Treponema* did not show statistically significant variation across groups (P > 0.05) (Fig. 2E). These abundance trends were generally consistent at the species level within each genus (Fig. 2F), and were reflected in site-specific patterns of species prevalence. Some taxa were ubiquitous, whereas others exhibited marked gradients in prevalence across populations.

We further examined species richness within key genera contributing to intergroup differences (Fig. 2F). Genera such as *Segatella, Faecalibacterium*, and *Prevotellamassilia* showed consistent species richness (100%) across all sites. In contrast, *Bacteroides, Treponema, Blautia, Phocaeicola*, and *Prevotella* displayed substantial variation in species richness (33–100%), while *Bifidobacterium, Ruminococcus*, and *Roseburia* showed moderate fluctuations (80–100%). Within the Irula population, species richness was particularly low for *Phocaeicola* (33.33%), *Bacteroides* (46.15%), and *Prevotella* (56.25%), but notably high for *Treponema*. In contrast, the Jenu Kuruba population exhibited high richness in *Bifidobacterium, Blautia, Prevotella*, and *Roseburia* (average >93%). The Konda Savara group showed elevated species richness (>94%) in *Bacteroides, Phocaeicola*, and *Ruminococcus*.

Interestingly, despite the considerable geographical distance between Chenchu and Jenu Kuruba, both forager populations residing in forested rural environments, their microbial profiles showed notable similarity. Specifically, the relative abundances of *Segatella, Prevotella, Phocaeicola*, and *Prevotellamassilia* were comparable between individuals from these groups, although the genera exhibited statistically significant intergroup differences. On the other hand, *Bifidobacterium, Blautia*, and *Roseburia* were consistently abundant in both Irula and Kurumba populations, possibly reflecting shared lifestyle practices involving agriculture and wage-based labour. While population identity and subsistence mode are key factors shaping the gut microbiome, our findings suggest that they alone do not fully explain the microbial variation across communities. Environmental and lifestyle heterogeneity within rural and urban contexts also play crucial roles. Overall, the site of residence emerged as the most dominant determinant of gut microbial composition in this cohort.

**Fig. 3:**
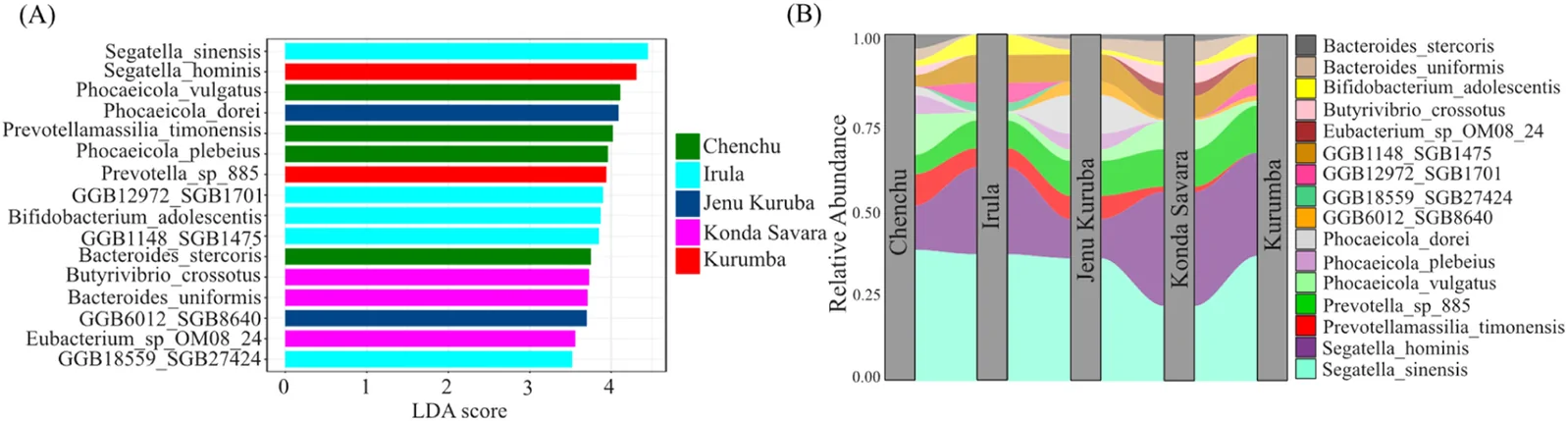
Differentially abundant microbial signatures across studied populations. Panel A presents the results of linear discriminant analysis effect size (LEfSe), identifying microbial taxa that exhibit significant differential abundance across populations (FDR-adjusted P < 0.05, LDA score ≥ 3.5). Bar length represents the corresponding LDA score, indicating the effect size of each discriminative taxon. Panel B displays the relative abundance of the differentially abundant taxa identified by LEfSe across the studied populations, highlighting their proportional representation within each microbial community and underscoring the distinct taxonomic contributions to population-level variation.

To further elucidate inter-population divergences in gut microbial composition, we employed Linear Discriminant Analysis Effect Size (LEfSe), a supervised method that integrates statistical significance with effect-size estimation to identify differentially abundant taxa. LEfSe identified 16 microbial taxa exhibiting significant differential abundance across the five tribal populations (FDR-adjusted P < 0.05, LDA score ≥ 3.5; Fig. 3A). The corresponding abundance patterns, visualized in Fig. 3B, encompassed both well-characterized commensals and poorly classified or unassigned taxa, highlighting the microbiome's taxonomic complexity across different ecological and cultural settings.

Among the most prominent findings, *Segatella sinensis* was markedly enriched in the Chenchu population, accounting for approximately 45% of its total gut microbial abundance. Its prevalence showed a progressive decline across Irula, Jenu Kuruba, Konda Savara, and Kurumba, suggesting a population-specific expansion potentially driven by localized dietary practices, environmental exposures, or genetic predispositions. Conversely, *Segatella hominis*, a closely related congener, exhibited an opposing distribution underrepresented in Chenchu but markedly more abundant in Konda Savara and Kurumba, suggesting micro-niche partitioning or divergent adaptive trajectories within the genus *Segatella*.

Additional population-specific trends were observed among members of the *Phocaeicola* genus. *Phocaeicola vulgatus* showed moderate abundance in Chenchu and Konda Savara but declined in Irula and Kurumba. *Phocaeicola dorei*, in contrast, displayed striking localization, attaining a relative abundance of 8.2% in Jenu Kuruba while being virtually absent in the other populations. *Phocaeicola plebeius* was exclusively identified in Chenchu and Jenu Kuruba, pointing to a restricted biogeographical distribution potentially influenced by group-specific ecological niches or subsistence strategies.

Similarly, *Prevotellamassilia timonensis* was enriched in both Chenchu and Jenu Kuruba, but was completely absent from the Kurumba microbiome. This discontinuous presence implies community-level factors influencing colonization, such as food fermentation practices, antibiotic exposure, or host-microbe interactions. In contrast, *Prevotella sp. 885* was detected across all five populations, though it reached its highest abundance in Kurumba. This broader yet variable distribution suggests that while some taxa maintain cosmopolitan presence, their relative success may be modulated by context-specific factors.

Functionally relevant commensals also demonstrated population-specific enrichment. *Bifidobacterium adolescentis*, a saccharolytic species implicated in host carbohydrate metabolism, was present in all five groups but showed peak abundance in Irula and Kurumba, potentially reflecting commonalities in plant-based or fiber-rich diets. *Bacteroides uniformis* was most prominent in Konda Savara, whereas *Bacteroides stercoris* was confined to Chenchu and Konda Savara. *Butyrivibrio crossotus*, a known butyrate producer, exhibited restricted presence, largely limited to Konda Savara, with minimal representation in Kurumba and was absent in other groups. Furthermore, *Eubacterium* sp. OM08-24 was detected only in the Konda Savara population, suggesting a population-specific distribution.

Collectively, these findings delineate a structured and ecologically nuanced portrait of gut microbiome composition across diverse tribal populations. The emergence of both widespread and highly localized taxa underscores the interplay between environmental exposures, cultural practices and potentially dietary habits in shaping microbial community structure.

**Fig. 4:**
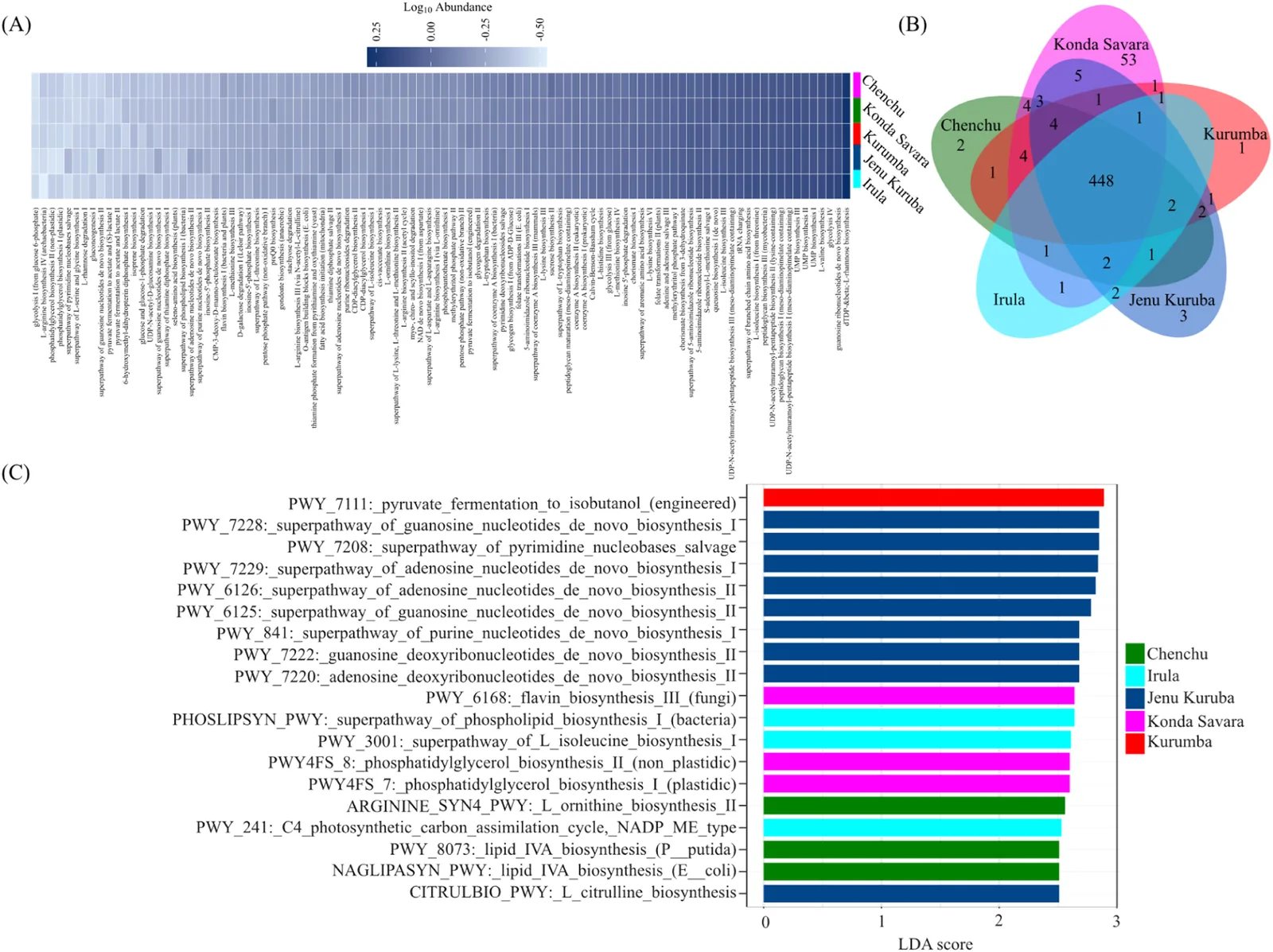
Functional landscape of the gut microbiome across studied tribal cohorts. Panel A presents a heatmap of the top 100 most abundant functional pathways, illustrating the community-wide distribution of microbial metabolic capacities across populations. Panel B depicts a Venn diagram summarizing the presence–absence-based overlap of functional signatures among cohorts, highlighting both shared and population-specific functional potentials. Panel C displays differentially enriched metabolic pathways identified across populations using LEfSe analysis (FDR-adjusted P < 0.05, LDA score ≥ 2.5), shown as a horizontal bar plot, reflecting functional stratification linked to population-specific microbiome configurations.

Human gut microbial communities perform essential metabolic functions critical to host health, including nutrient processing, immune modulation, and the metabolism of dietary compounds (nutritional adaptation). While taxonomic profiles reveal microbial identities, functional profiling provides insights into their biochemical potential. Here, we investigated the functional potential of gut microbiota across populations with distinct ecological contexts and subsistence strategies. Functional profiling was conducted by quantifying the relative abundances of 546 microbial metabolic pathways annotated through the MetaCyc database, a curated repository of experimentally validated metabolic pathways (Supplementary File S9). The top 100 most abundant pathways are visualized in Fig. 4A. To assess functional differentiation among populations, we first compared pathway occurrence patterns across populations (Fig. 4B), followed by Linear Discriminant Analysis Effect Size (LEfSe), which identifies differentially abundant features by integrating statistical significance with effect size. Among the identified pathways, a substantial subset (n = 448) was consistently present at high levels across all populations, suggesting the existence of a conserved functional core. This included essential microbial functions such as glycolysis III (from glucose), tRNA charging, and folate transformations (E. coli variant), which are central to energy metabolism, protein synthesis, and cofactor biogenesis. These shared pathways underscore the foundational metabolic architecture of the gut microbiome, irrespective of host population.

In contrast, population-specific deviations were evident, with certain pathways showing marked variation in abundance, indicating differences in the predicted functional potential of gut microbial communities. Top 20 most variable pathways (Supplementary Fig. 7) revealed distinct patterns of metabolic specialization. Glycolysis-related and L-methionine biosynthesis pathways showed uniformly high abundance, indicating conserved energy metabolism, whereas nucleotide biosynthesis, rhamnose biosynthesis, and queuosine biosynthesis pathways displayed population-specific enrichment. For instance, while glycolysis III maintained uniformly high abundance, reinforcing its role as a core metabolic pathway, other functions such as “4-hydroxyphenylacetate degradation” and “1,2-dichloroethane degradation” displayed population-specific enrichment, particularly in Jenu Kuruba and Kurumba, suggesting variation in the microbial genetic potential for xenobiotic degradation (Supplementary File S9). These differences may be associated with variation in dietary or environmental exposures.

Furthermore, amino acid biosynthetic pathways such as L-tryptophan and L-valine synthesis exhibited differential distribution, indicating variation in microbial functional potential related to amino acid metabolism that could be shaped by host diet and nutritional status. LEfSe identified 19 discriminatory metabolic pathways enriched across the five populations (FDR-adjusted P < 0.05, LDA score ≥ 2.5), indicating population-specific functional differentiation (Fig. 4C).

In the Irula population, significant enrichment of xenobiotic degradation pathways, such as those targeting 1,2-dichloroethane and other aromatic compounds, may be consistent with differences in environmental exposures or dietary practices. The Jenu Kuruba microbiome showed notable enrichment in mucin degradation and short-chain fatty acid (SCFA) production, indicating a greater abundance of pathways associated with host–microbiota metabolic interactions. In Kurumba, elevated pathways for the metabolism of aromatic amino acids and phenolic compounds may be consistent with dietary characteristics rich in plant-derived secondary metabolites. The Chenchu population exhibited significant enrichment in amino acid biosynthesis pathways, including those for L-arginine, L-tryptophan, and L-valine, possibly compensating for a protein-scarce diet. Meanwhile, the Konda Savara microbiome demonstrated higher representation of pathways involved in lipid metabolism and ureide biosynthesis, which may be associated with differences in dietary composition (dietary lipid utilization) or microbial nitrogen metabolism. Pathways were broadly classified into functional categories, including central carbon metabolism, amino acid biosynthesis, lipid metabolism, xenobiotic degradation, SCFA production, and nucleotide cofactor biosynthesis. These categories may serve as biomarkers of population-specific metabolic potential but also reflect ecological and cultural differences influencing microbial community function.

The findings reveal that each tribal population harbours a microbiome with distinct predicted functional profiles. Enrichment of host-interactive pathways, including those involved in mucin degradation and short-chain fatty acid (SCFA) production, suggests differences in the functional potential of gut microbial communities and may indicate population-specific host–microbiota interactions. Despite this divergence, the consistent presence of pathways underpinning core microbial metabolism highlights a stable and conserved functional backbone of the human gut ecosystem.

Collectively, this in-depth functional metagenomic analysis underscores the dynamic interplay between host ecology and microbial function. It provides crucial insights into how traditional and transitional lifestyles shape gut microbial metabolism and offers a foundation for understanding adaptive trajectories and microbial resilience in diverse indigenous populations.

**Fig. 5:**
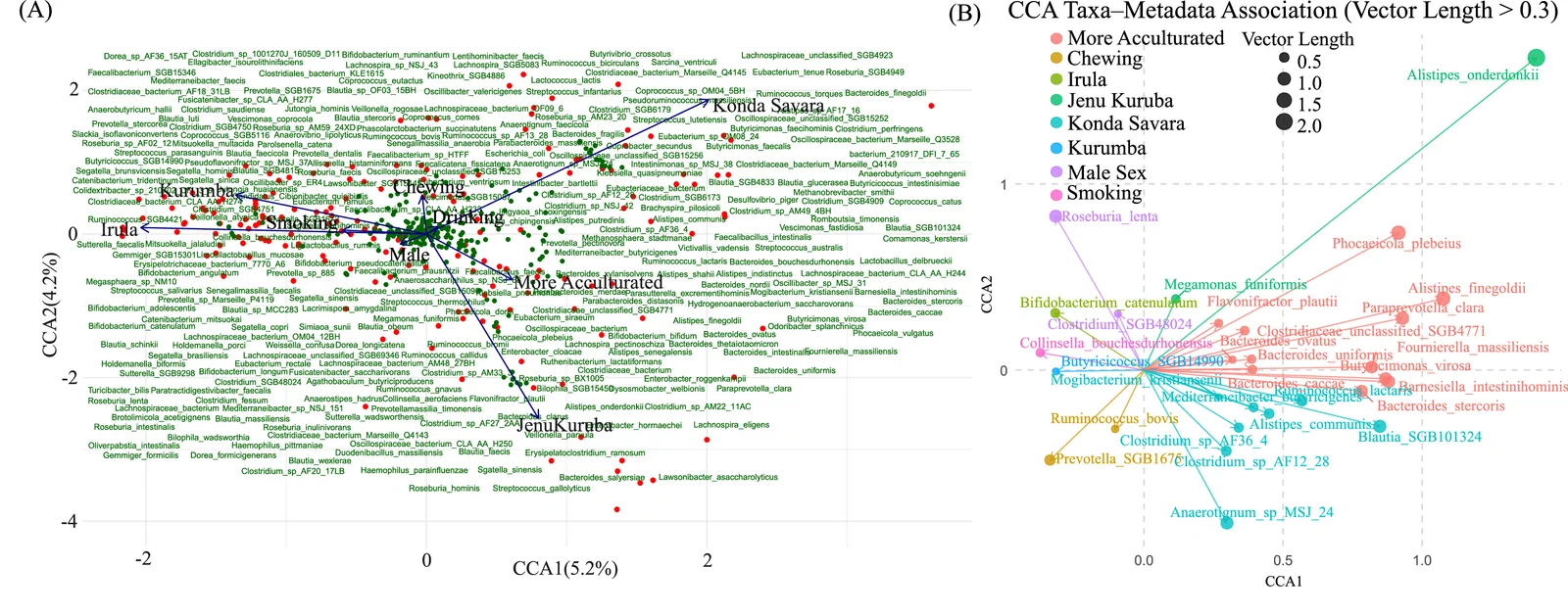
**Canonical Correspondence Analysis (CCA) of gut microbial taxa constrained by lifestyle-associated variables.** Panel A CCA ordination plot based on the abundance profiles of 489 gut-associated taxa, constrained by lifestyle habits and practices across populations. Axis 1 and Axis 2 explain 6.5% and 5.3% of the variance, respectively. Panel B highlights lifestyle-associated variables significantly associated with microbial community composition are shown as vectors. Arrow length indicates the relative strength of the association with community variation, and only variables with vector lengths >0.3 are displayed.

Canonical Correspondence Analysis (CCA) demonstrated that gut microbial composition was significantly associated with the evaluated explanatory variables (overall permutation test: χ² = 0.216, F = 3.96, P = 0.001) (Fig. 5A and Supplementary Fig. 6). The first two canonical axes explained 9.18% of the constrained variation, with CCA1 accounting for 5.30% and CCA2 accounting for 3.88%. Permutation tests indicated that the first five canonical axes were statistically significant (CCA1-CCA4: P = 0.001; CCA5: P = 0.031), whereas the remaining axes were not significant, indicating that the major interpretable structure was captured by the leading canonical dimensions (and Supplementary Fig. 6). Among the populations analyzed, the Jenu Kuruba group emerged as the most functionally and taxonomically distinct. This was evidenced by the strong alignment of key microbial taxa, most notably *Alistipes onderdonkii*, with the vector representing this population in the ordination space. Within the ordination space, the Jenu Kuruba population was most strongly associated with *Alistipes onderdonkii*, which exhibited the longest species vector (2.19) and was primarily aligned with CCA2, indicating that this species contributed strongly to the compositional differentiation of this population. This taxon has been associated with host lipid metabolism and immune modulation, suggesting that the Jenu Kuruba harbor a distinct gut microbial composition that may be associated with their dietary practices, ecological setting, or cultural isolation. The position and orientation of this vector along CCA2 are consistent with differences in gut microbiome composition observed in this group (Fig. 5B). The Konda Savara population also demonstrated a moderately strong microbial signature, primarily oriented along CCA1. The ordination revealed a distinct alignment with *Blautia SGB101324*, a taxon known for its role in anaerobic carbohydrate metabolism, particularly the production of short-chain fatty acids (SCFAs) and maintenance of mucosal health. This suggests that Konda Savara may possess a gut microbial profile enriched for beneficial anaerobes, potentially influenced by a traditional, fiber-rich plant-based diet.

In contrast, the Chenchu population exhibited a relatively weaker vector gradient, with limited direct alignment of dominant microbial species. However, partial alignment was observed with taxa from the *Ruminococcaceae* family, generalist anaerobes associated with a broad range of dietary substrates. This diffuse pattern implies a less specialized microbial structure, possibly arising from ecological generalism or transitional exposure to diverse dietary and environmental factors. The Kurumba population displayed a mild to moderate vector structure. While no single dominant taxon was highly aligned, the microbial community appeared to trend toward taxa such as *Paraprevotella* and *Prevotella* species, which are known for mucin degradation and aromatic compound metabolism. These functional attributes suggest microbial adaptation to niche-specific substrates, potentially resulting from semi-acculturated dietary shifts or environmental exposures characteristic of this group.

The Irula population showed the least distinct microbial vector gradient among all groups analysed. There was minimal or no strong alignment with taxa identified only in the Irula population within the current dataset. This pattern may indicate a greater degree of microbial homogenization or ecological generalism, potentially reflecting increased contact with other communities or reduced cultural isolation. Alternatively, the absence of a distinct microbial signature in the Irula population may reflect overlapping ecological exposures or convergence in diet and lifestyle with neighbouring groups.

Taken together, the significant CCA model (F = 3.96, P = 0.001), the first two canonical axes explaining 9.18% of the constrained variation, and the observed species-vector alignments indicate that population identity and lifestyle-related variables jointly contribute to gut microbial structuring. Although the proportion of explained variation was modest, the highly significant permutation tests support that these associations are unlikely to have arisen by chance. The distinct gradient observed from the highly specialized microbiota of Jenu Kuruba to the generalized, less differentiated microbial community of Irula reflects varying degrees of microbial specialization, ecological isolation, and host–microbiome co-adaptation across these tribal populations.

Additionally, certain species, such as *Phocaeicola plebeius* and *Alistipes finegoldii,* aligned more strongly with sociocultural vectors such as acculturation level (Fig. 5B). These taxa may serve as microbial indicators of cultural transitions, further reinforcing the influence of both environmental and cultural factors on microbial community structure. The robustness of these associations is supported by the strong eigenvalues of the canonical axes and the highly significant permutation P-values (Supplementary Fig. 6). These results affirm that the observed microbial distributions are not artifacts of random variation but are statistically significant outcomes of complex interactions between host identity, lifestyle, and ecological exposure.

### Candidate keystone taxa identification

A defining feature of microbiome taxa associated with host health and lifestyle is their consistent prevalence and strong structural integration within the gut microbial communities of non-diseased individuals. Our analysis reveals that such microbial signatures are highly community-specific, particularly among populations adhering to traditional lifestyles with minimal exposure to Westernized dietary practices and unregulated medical interventions. These populations offer a unique perspective into the composition of a healthy gut microbiome shaped by long-standing ecological and cultural traditions. We define this feature as a core association, where “core” denotes widespread prevalence across individuals, and “association” reflects strong compositional influence within the broader microbial community. This is quantified through co-occurrence and community association metrics, as detailed below. Taxa exhibiting both high prevalence and strong community associations are considered foundational constituents of a stable and healthy gut microbiome. Perturbations in these taxa often correlate with broader shifts in microbiome structure, implicating them as potential drivers or indicators of ecosystem stability or dysbiosis.

**Fig. 6:**
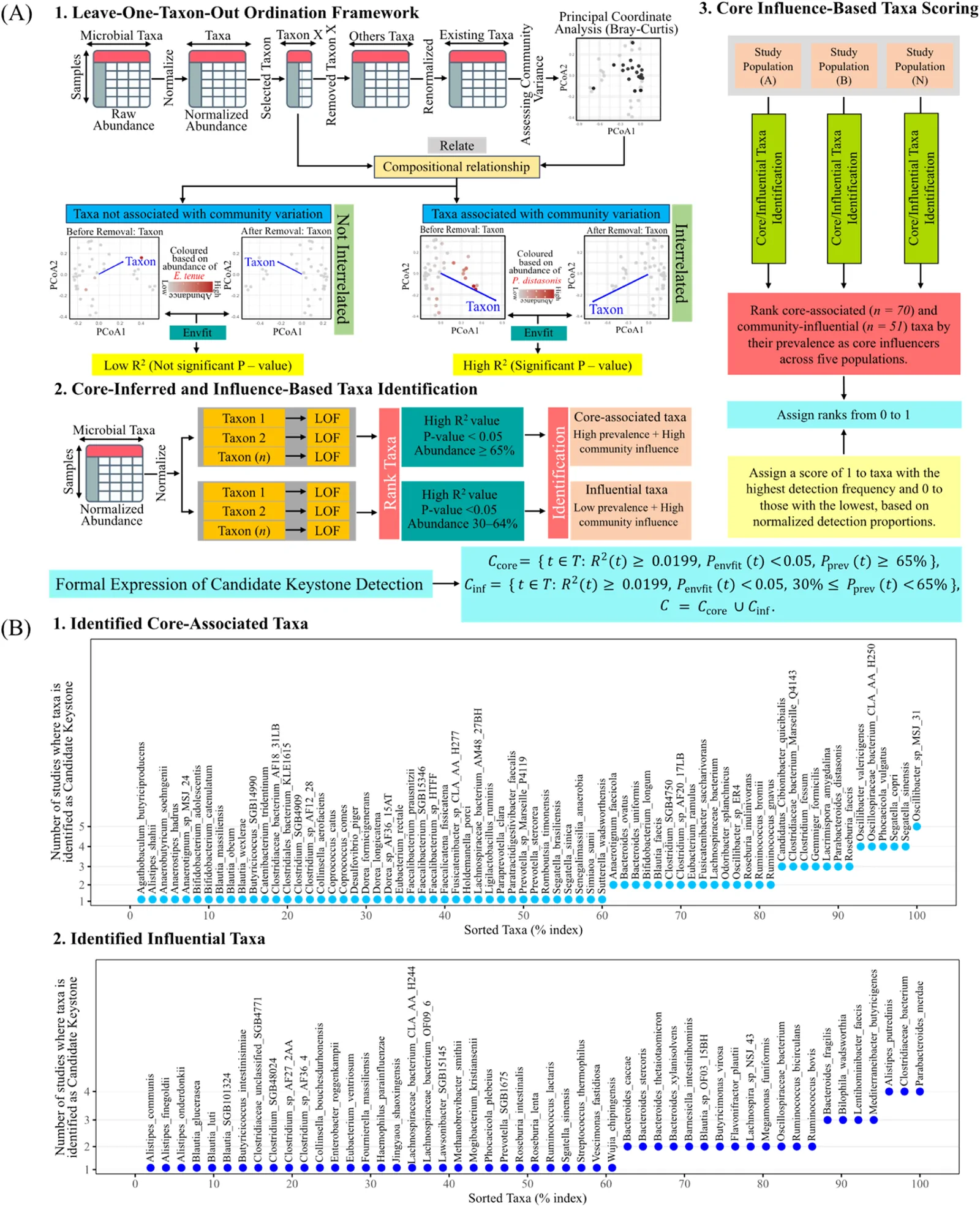
**Core-association analysis of gut microbial taxa across five study populations using the leave-one-taxon-out ordination framework.** A. Schematic overview of the analytical workflow employed to identify core-associated microbial taxa within each population. To evaluate the degree of community-level influence exerted by a given taxon X in a cohort (population), the abundance of X was first excluded from all microbiomes within the cohort. The resulting taxonomic profiles were then renormalized, and beta-diversity metrics (Bray-Curties) were recomputed using Principal Coordinate Analysis (PCoA). Subsequently, the variation in community composition captured by PCoA axes was regressed against the original abundance of X using the envfit function. A strong statistical association (i.e., high *R²* and low *P*-value) indicates that the removed taxon substantially influenced the structure of the remaining community. As illustrated in the example involving *P. distasonis* and *E. tenue*, taxa with significant *R²* values (*P* < 0.05) and high prevalence (≥65%) were designated as core-associated taxa, while those with moderate prevalence (30–64%) were classified as core-influential taxa. Taxa fulfilling both criteria were designated as candidate Keystone taxa, reflecting their consistent and strong influence on gut community architecture across the studied populations. B. Ranked list of gut-associated taxa based on their recurrence as core-associated or core-influential taxa across the five cohorts (populations). Taxa are ordered in descending frequency of detection, providing a robust consensus score that highlights their cross-population relevance and consistent association with community structure within the gut microbiome.

**Fig. 7:**
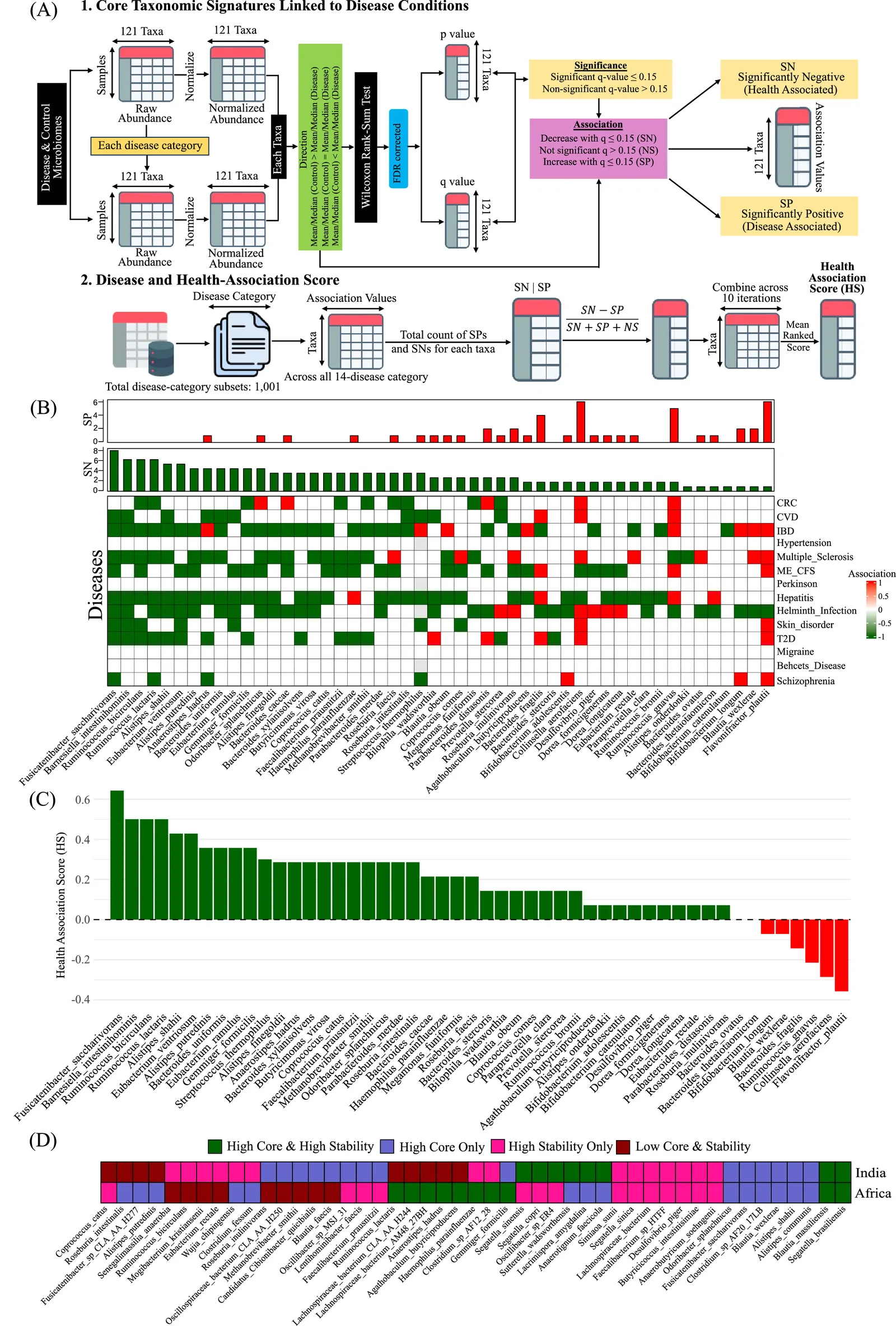
**Computation of health-association scores (HS) and cross-population validation of 121 candidate keystone taxa.** Panel A illustrates the workflow used to evaluate taxon–disease associations using the Wilcoxon rank-sum test by comparing disease and healthy control samples, thereby identifying alteration patterns of the 121 candidate keystone taxa within each disease category. Based on the direction and statistical significance of the association, each taxon was classified as SP (significantly positive; disease-associated), SN (significantly negative; health-associated), or NS (not significant). To evaluate the robustness of health associations, all 1,001 unique combinations of 10 out of the 14 disease categories were exhaustively evaluated. HS values were calculated for each subset, and the final HS for each taxon was obtained as the mean across all subset-specific estimates. Panel B shows the disease-association matrix for each candidate keystone taxon across the 14 disease categories. Taxa were classified as SP (disease-associated), SN (health-associated), or NS (not significant) according to the direction of differential abundance and the FDR-adjusted significance threshold (q ≤ 0.15). The corresponding SP and SN counts used for HS calculation are also shown. Panel C presents the final Health-Association Scores (HS), calculated as the mean across all 1,001 unique disease-category subsets, with higher positive values indicating more consistent associations with healthy controls and lower negative values indicating more consistent associations with disease. Panel D depicts the cross-population stability of candidate keystone taxa, illustrating the reproducibility and robustness of conserved taxa across the studied tribal populations.

To identify a robust microbial signature reflective of gut health, we implemented a three-step analytical framework. First, we identified taxa that were both widely prevalent and structurally influential across populations and designated them as candidate keystone taxa based on their community-level association scores (Methods Section and Fig. 6). Second, we cross-referenced these candidate keystone taxa with global disease association databases to refine a candidate set of health-associated microbial taxa (Methods Section and Fig. 7). Finally, we conducted microbial co-occurrence network analysis to further consolidate and visualize the structural roles of these taxa within the gut ecosystem, thereby elucidating their contribution to community organization and resilience.

To identify putative healthy core candidate keystone taxa, we implemented a preliminary prevalence-based screening at the species-level resolution within each community. Taxa were retained if they exhibited an abundance of ≥1 read in at least 20% of individuals within a given community, ensuring their structural persistence and relevance. Following this initial filtering, a refined subset of 489 taxa previously defined as “gut-associated” based on their cumulative contribution to intergroup dissimilarity was selected for downstream analysis across the entire study cohort. This subset was derived from SIMPER statistical analysis, where taxa contributing cumulatively to 91% of the average community dissimilarity were prioritized (Justification in Supplementary Fig. 14).

Taxon prevalence was defined as the proportion of individuals in which a taxon was detected. Community-level structural influence was assessed using a leave-one-taxon-out ordination framework (Methods Section and Fig. 6), in which taxon abundance was correlated with PCoA-based community structure using the envfit function. A taxon was considered to exert a meaningful structural influence if it exhibited a strong association with the PCoA ordination axes (R²) and met a statistical significance threshold (P < 0.05), indicating a consistent contribution to community-level variation. This procedure was systematically applied to all 489 candidate taxa across the five study cohorts (Methods Section and Fig. 6A).

For each taxon, we quantified community-association strength using the envfit-derived R² and P-values. Taxa that were significantly associated (P < 0.05) and present in at least 65% of individuals within a cohort were classified as ‘core-associated’. Those that met the same significance threshold but were present in at least 30% of individuals were defined as ‘core-influential’ taxa (Methods Section and Fig. 6A). This analysis was independently repeated for each of the five study cohorts. To assess cross-cohort consistency, taxa were then ranked by their recurrence as core-associated or core-influential across cohorts. For each taxon, we computed a ‘core-association’ and ‘core-influence’ score, reflecting the number of populations in which it was classified as significant. Taxa with consistently high scores across populations were designated as candidate keystone taxa (Fig. 6B1, 6B2, and Supplementary Table 9), indicating their persistent association with gut microbial community structure across populations.

A total of 121 candidate keystone taxa were identified across the study populations, comprising 70 core-associated and 51 core-influential taxa. The majority of these taxa (73 of 121; 60%) were exclusive to a single community, while 28 taxa (23%) were detected in two communities, 11 (9%) in three communities, and 8 (7%) in four communities. Only a single taxon was found to be consistently present across all five studied communities (Fig. 6B1, 6B2). Notably, the subset of 48 taxa found in more than one population accounted for over 65% of the core-associated taxa in more than 80% of the studied cohorts, indicating that the property of core association is largely restricted to a relatively small and recurrent fraction of the gut microbiome.

Among these, the nine most consistently detected core-associated taxa included three *Oscillibacter* spp. and two *Segatella*-*Bacteroides* species, alongside *Phocaeicola vulgatus, Alistipes putredinis, Clostridiaceae bacterium*, and *Parabacteroides merdae*. Given that our analysis is based on gut microbial profiles from non-diseased individuals, it is important to emphasize that the designation of core-associated or influential taxa does not imply inherent health-promoting functionality. Some of these taxa may be implicated in disease states, suggesting that structural centrality (i.e., core status) is not synonymous with beneficial roles in host physiology.

Furthermore, core association was not necessarily linked to high relative abundance. Several low-abundance taxa (<1% relative abundance) demonstrated strong and consistent community-level associations (R², P < 0.05) and high prevalence (≥65%) across individuals. Representative examples of such core-associated but low-abundance taxa include *Candidatus Cibionibacter quicibialis, Clostridiaceae bacterium Marseille-Q4143, Clostridium fessum, Gemmiger formicilis, Lacrimispora amygdalina*, and *Parabacteroides distasonis*. Among all identified core taxa, only *Roseburia faecis* exhibited a relative abundance greater than 1%.

Similarly, influential taxa defined by significant community association (R², P < 0.05) and moderate prevalence (30–64%) included *Bacteroides fragilis, Bilophila wadsworthia, Lentihominibacter faecis*, and *Mediterraneibacter butyricigenes*, in addition to several of the top-ranked community-associated taxa. These findings underscore that core or influential status reflects structural consistency and structural integration within the microbial community rather than abundance alone or direct association with health. Nevertheless, given the potential for overlapping roles in health and disease, we subsequently performed a global disease-association analysis of the identified core-associated and influential taxa to further evaluate their relevance in pathological contexts.

### Health association of the identified candidate keystone taxa

To investigate the potential health relevance of gut microbiome members previously identified as candidate keystone taxa (defined as core-associated and influential), we performed a targeted disease association analysis across a wide range of human disease conditions. Candidate keystone taxa, characterized by their consistent presence and disproportionate association with community structure within healthy individuals, are often presumed to play beneficial roles; however, such assumptions remain unverified. Structural ubiquity does not necessarily equate to functional advantage or health-promoting capacity. Some taxa may be critical for microbial network integrity or consistently present across individuals, yet may exhibit distinct behaviour under pathological conditions. To address this uncertainty, we conducted a comprehensive, hypothesis-driven association study aimed at determining whether these taxa show systematic enrichment or depletion in disease states.

We compiled a dataset comprising 5,626 individuals from 21 independent cohorts, including both diseased and non-diseased (control) individuals. These cohorts spanned 18 distinct disease types, which were systematically grouped into 14 broader disease categories to ensure analytical consistency and adequate statistical power. A stepwise strategy guided cohort selection, prioritizing balanced case-control representation and minimizing overrepresentation of any single disease type. This approach reduced sampling bias and ensured equitable analytical weight across disease categories.

For analysis, all cohorts within a given disease category were aggregated into a unified dataset to enable cross-cohort harmonization while preventing overrepresentation from categories with a higher number of contributing cohorts. Individuals within each aggregated dataset were stratified into disease-positive and disease-negative groups. Differential abundance analysis was performed using the Wilcoxon rank-sum test, a robust, non-parametric method well-suited for microbiome data due to its resilience to non-normal distributions and its ability to detect shifts in taxon abundance.

Taxa showing significantly higher abundance in disease-negative individuals were classified as health-associated, while those enriched in disease-positive individuals were classified as disease-associated. This analysis was conducted independently for each of the 14 disease categories, allowing the identification of microbial taxa consistently associated with either health or disease across a diverse spectrum of clinical conditions (Fig. 7A). Taxa enriched in control individuals are positioned on the left side of the visualization, indicating potential health-supporting roles, whereas taxa enriched in disease are placed on the right, suggesting a potential link to dysbiosis or opportunistic proliferation under pathological stress (Fig. 7B).

The visualization uses a colour gradient to depict the strength and direction of association, with cooler tones denoting enrichment in controls and warmer tones indicating disease enrichment. Taxa consistently associated across multiple disease categories, particularly with concordant trends, are highlighted by SN (Significantly Negative; health-associated) and SP (Significantly Positive; disease-associated) counts (Fig. 7B). Furthermore, taxa were organized along a graded spectrum based on the consistency of their health-association patterns. This was quantified through a bootstrapped, iteration-based health-association score (HS) (Figures 7B and 7C) for the 121 identified candidate keystone taxa (combined core-associated and influential taxa).

The analysis revealed that 71 of the 121 taxa (58%) were not consistently associated with either disease or non-disease states. In contrast, 50 taxa demonstrated consistent associations, either enriched or depleted across disease and non-disease categories (Fig. 7C). Among these, 44 taxa exhibited recurrent enrichment in non-diseased individuals, while 6 were consistently enriched in disease contexts. Notably, taxa such as *Fusicatenibacter saccharivorans, Barnesiella intestinihominis, Ruminococcus bicirculans, Ruminococcus lactaris*, and *Alistipes shahii* showed strong, recurrent enrichment in healthy individuals.

Meanwhile, several taxa, including *Bacteroides xylanisolvens, Butyricimonas virosa, Coprococcus catus, Faecalibacterium prausnitzii, Bacteroides ovatus, Bacteroides thetaiotaomicron*, and *Methanobrevibacter smithii*, exhibited non-significant trends across both groups but were more frequently enriched in control samples across multiple disease conditions, suggesting a potential role in maintaining ecosystem stability or host homeostasis. In contrast, a subset of influential taxa, despite their associations with community structure, were repeatedly enriched in disease states. These included *Bifidobacterium longum, Blautia wexlerae, Bacteroides fragilis, Ruminococcus gnavus, Collinsella aerofaciens*, and *Flavonifractor plautii*, which may act as general indicators of dysbiosis or reflect condition-specific microbial shifts that compromise community integrity.

Building on these observations, the cross-cohort reproducibility of core candidate keystone taxa identified in Indian tribal populations was evaluated using an independent African tribal cohort. Of the 121 core candidate keystone taxa identified in the Indian cohorts, 47 were reproducibly detected in the African cohort (38.8%), indicating partial conservation of candidate keystone microbial signatures across geographically distinct traditional populations (Fig. 7D and Supplementary Fig. 15). To further evaluate the reproducibility of community stability, stability scores of these reproducible candidate keystone taxa were compared between the Indian discovery cohort and the African validation cohort. Stability scores showed a significant positive correlation across cohorts (Spearman's ρ = 0.259, P = 0.00226), indicating overall reproducibility of candidate keystone stability despite geographic separation. Taxa exhibiting the smallest cross-cohort ranking differences, including *Phocaeicola vulgatus, Segatella brasiliensis, Parabacteroides merdae, Segatella copri, Alistipes shahii, Fusicatenibacter saccharivorans, Segatella sinensis, Segatella sinica*, and *Lachnospiraceae* bacterium AM48-27BH, demonstrated the greatest concordance of stability rankings across the two cohorts. Within this reproducible subset, 15 taxa (31.9%) retained concordant categorical classification in both cohorts with respect to core prevalence and stability, reflecting consistent community-level associations despite substantial geographic separation. These concordant taxa were consistently classified as high core and high stability, high core only, or high stability only across both populations, suggesting reproducible patterns of prevalence and stability, whereas the remaining taxa exhibited cohort-specific categorical shifts, indicative of context-dependent variation in core prevalence or stability. Notably, 18 of the reproducible taxa also displayed consistent health associations, including key butyrate-producing and metabolically relevant commensals such as *Fusicatenibacter saccharivorans, Ruminococcus bicirculans, Faecalibacterium prausnitzii, Roseburia intestinalis*, and *Anaerostipes hadrus*, collectively supporting the presence of a reproducibly identified subset of gut microbial candidate keystone taxa with consistent community-level and health associations across traditional human populations (Supplementary Fig. 15 and Supplementary Tables S11 & S12).

**Fig. 8:**
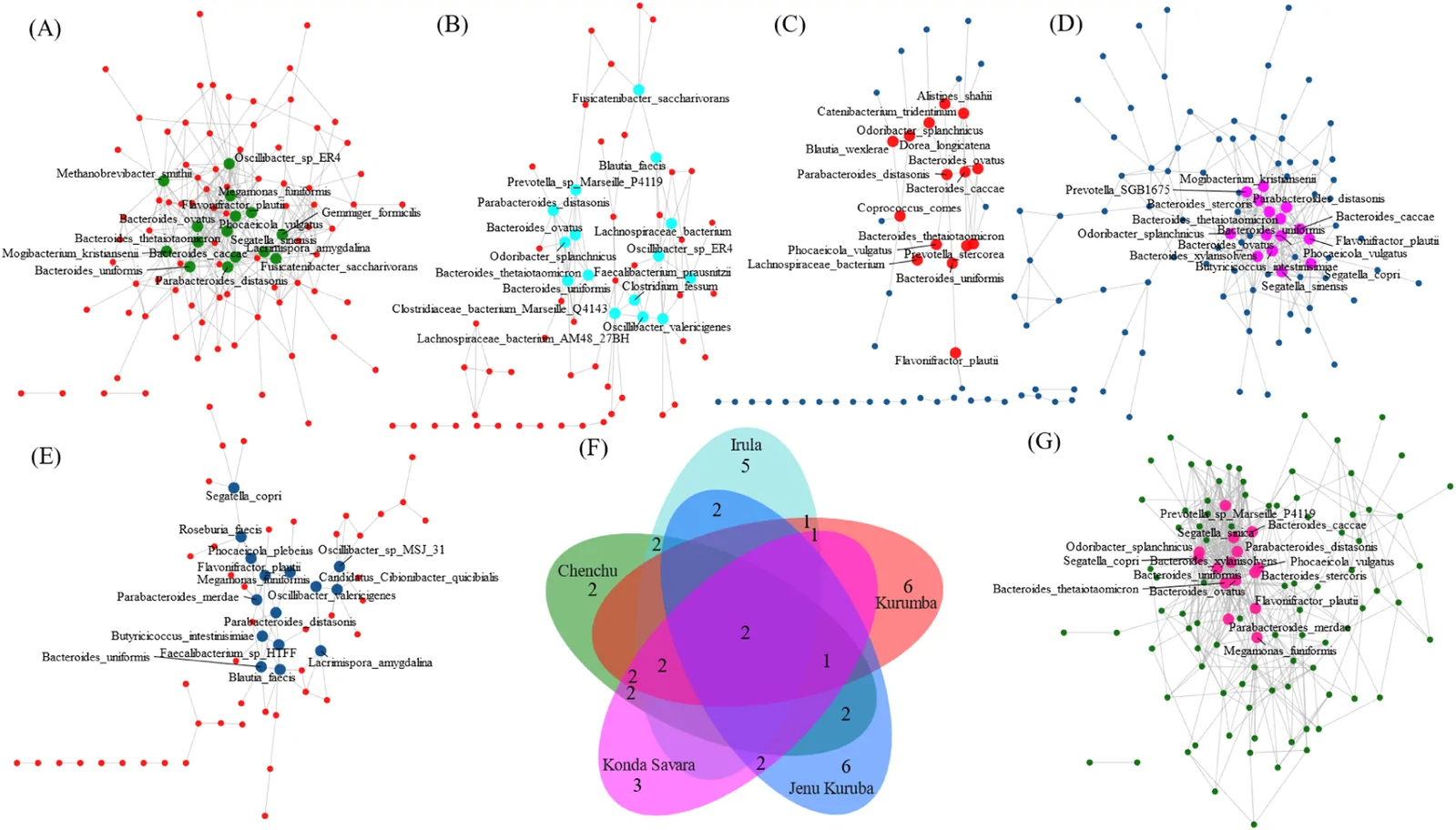
**Microbial co-occurrence networks of candidate keystone taxa across five tribal populations and the integrated community.** Networks were inferred from centred log-ratio (CLR)-transformed microbial abundance data using Pearson correlation analysis with Benjamini–Hochberg false discovery rate correction. Only significant associations satisfying an absolute correlation coefficient of |r| ≥ 0.30 and an adjusted *P* < 0.05 were retained. Nodes represent microbial taxa and edges represent significant positive or negative co-occurrence relationships. Taxa ranked among the top 15 according to degree, betweenness, and closeness centrality are highlighted to indicate structurally central members of each network. Panels A–E show the population-specific co-occurrence networks for Chenchu A, Irula B, Kurumba C, Konda Savara D, and Jenu Kuruba E, with labels displayed only for the top 15 taxa. Panel F presents the overlap of the top 15 structurally central taxa among the five populations. Panel G shows the integrated co-occurrence network constructed by combining all study populations, highlighting the top 15 structurally central taxa within the combined microbial community.

Following the filtered, target-specific analysis, we further investigated whether taxa associated with multiple disease conditions occupied structurally important positions within the gut microbial co-occurrence network. Using a statistically filtered correlation-based network, taxa were ranked according to degree, betweenness, and closeness centrality to identify the top 15 structurally central members of each population-specific network and the integrated microbial network (Fig. 8, Supplementary Fig. 10).

Comparison of the top-ranked taxa across population-specific networks revealed both conserved and community-specific patterns of network organization. *Bacteroides uniformis* and *Parabacteroides distasonis* were the only taxa consistently identified among the top 15 central taxa in all five tribal communities (Chenchu, Irula, Jenu Kuruba, Konda Savara, and Kurumba). *Bacteroides thetaiotaomicron* and *Bacteroides ovatus* were shared among four communities (Chenchu, Irula, Konda Savara, and Kurumba), whereas *Flavonifractor plautii, Bacteroides caccae*, and *Odoribacter splanchnicus* occurred among the top-ranked taxa in three communities. Several additional taxa, including *Phocaeicola vulgatus, Megamonas funiformis, Segatella copri, Segatella sinensis, Butyricicoccus intestinisimiae, Blautia faecis, Mogibacterium kristiansenii, Fusicatenibacter saccharivorans,* and *Oscillibacter* sp. ER4, were shared between two communities, whereas the remaining taxa were restricted to individual populations, demonstrating considerable population-specific variation in microbial network topology despite the presence of a conserved set of highly connected taxa (Fig. 8A–F).

To complement the population-specific analyses, we next examined the integrated microbial network comprising all study populations to determine whether the same taxa retained structurally central positions across the combined dataset (Fig. 8G). Among the six taxa previously identified as being associated with multiple disease conditions (*Bifidobacterium longum, Blautia wexlerae, Bacteroides fragilis, Ruminococcus gnavus, Collinsella aerofaciens*, and *Flavonifractor plautii*), only *Flavonifractor plautii* remained among the top 15 structurally central taxa in the integrated network. At the population level, *Flavonifractor plautii* was consistently represented among the highly central taxa in the Chenchu, Jenu Kuruba, Konda Savara, and Kurumba communities but was absent from the top-ranked taxa in the Irula network. In contrast, *Blautia wexlerae* exhibited population-specific centrality, occurring only in the Kurumba community. The remaining disease-associated taxa (*Bifidobacterium longum, Bacteroides fragilis, Ruminococcus gnavus*, and *Collinsella aerofaciens*) were not identified among the top-ranked taxa in either the integrated or any population-specific network, indicating that disease association and structural centrality represent distinct characteristics.

Analysis of interaction patterns further showed that all top 15 taxa in the integrated network exhibited both positive and negative significant correlations and were therefore classified as having dual interaction roles. Similar mixed interaction profiles predominated across the population-specific networks. In both the Chenchu and Konda Savara communities, all highly central taxa displayed dual interaction roles, indicating that each top-ranked taxon participated in both positive and negative association patterns. In contrast, a limited number of taxa exhibited community-specific interaction profiles. In the Irula community, *Blautia faecis* showed exclusively positive associations, whereas *Parabacteroides distasonis* exhibited only positive interactions in the Jenu Kuruba community. The Kurumba network displayed the greatest deviation from the integrated pattern, where *Bacteroides thetaiotaomicron, Blautia wexlerae*, and *Flavonifractor plautii* exhibited exclusively positive associations, while *Prevotella stercorea* showed exclusively negative associations. Despite these population-specific differences, dual interaction profiles predominated across all networks, indicating that most highly connected taxa participated in both positively and negatively correlated association patterns rather than being restricted to a single interaction type (Supplementary Fig. 10).

Overall, the integrated and population-specific network analyses identified a conserved core of highly connected taxa shared across multiple tribal populations together with numerous population-specific central taxa. While *Flavonifractor plautii* was the only disease-associated taxon that consistently occupied structurally central positions across several communities and in the integrated network, the remaining disease-associated taxa exhibited limited or no network prominence. Collectively, these findings demonstrate that structural centrality and disease association capture different characteristics of microbial taxa and provide an association-based view of microbial community organization across diverse tribal gut microbiomes. As the networks were inferred from statistically filtered correlations of cross-sectional abundance data, the observed associations should be interpreted as co-occurrence patterns rather than direct ecological interactions or causal relationships.

Altogether, this analysis deepens our understanding of the gut microbiome by revealing how structural importance, taxonomic persistence, and health relevance intersect. It provides a valuable foundation for future functional validation and potential clinical translation of key microbial taxa.

**Fig. 9:**
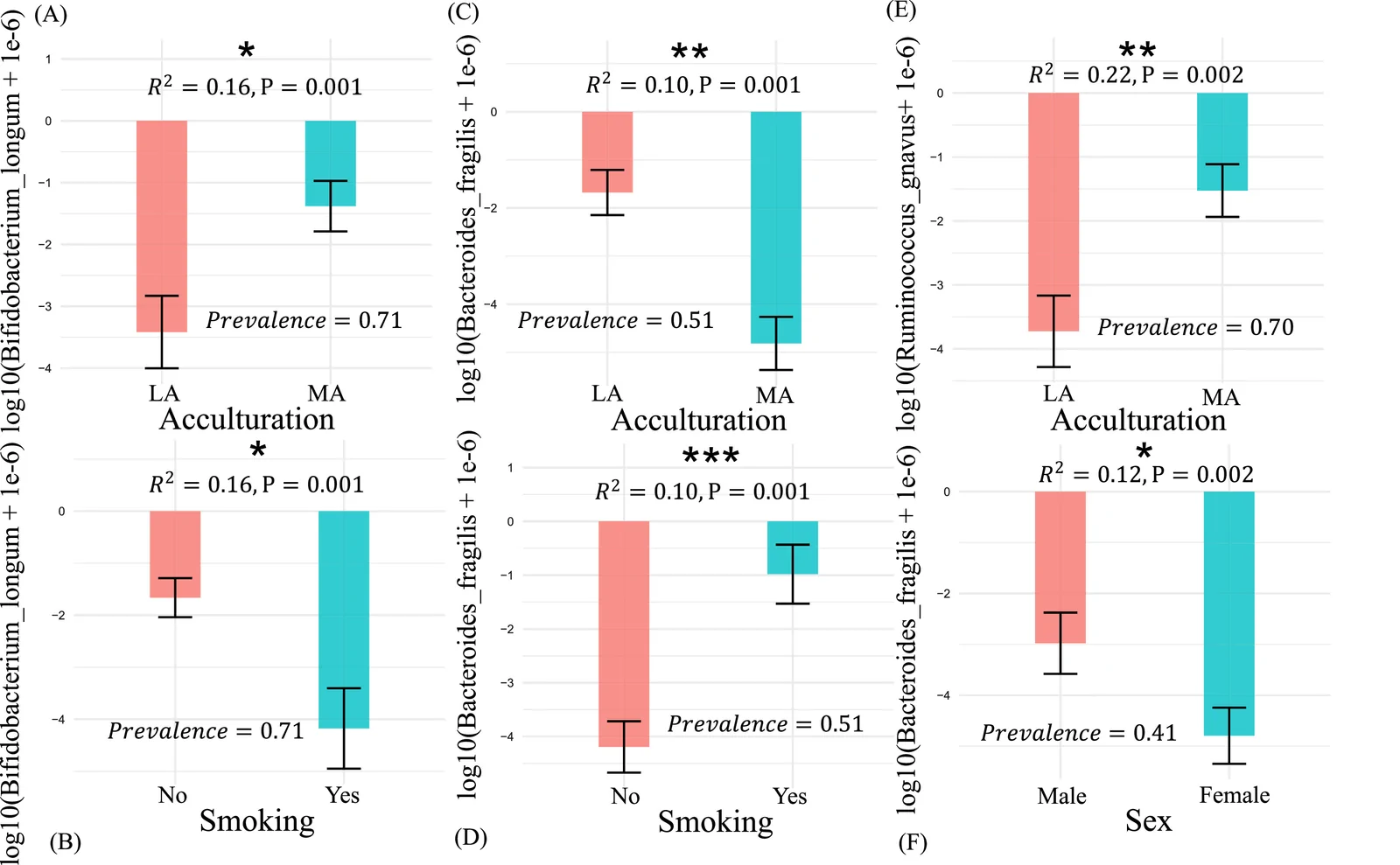
**Population-specific associations between disease-associated taxa and lifestyle-related variables.** Population-specific significance (P < 0.05) testing of disease-associated taxa with lifestyle-associated factors, based on log10-transformed abundance values. Panels A–B depict statistically significant associations in the Chenchu population, along with corresponding community association values. Panels E and F represent the significant associations for the Jenu Kuruba and Kurumba populations, respectively.

To explore the influence of lifestyle-induced factors on the distribution of disease-associated taxa, we observed that *Flavonifractor plautii* and *Bifidobacterium longum* exhibited significant (P < 0.05) differences in abundance across populations. For instance, in pairwise comparisons, *Flavonifractor plautii* showed a stronger level of significance between Jenu Kuruba and Kurumba (P < 0.01), followed by Jenu Kuruba and Konda Savara, and Chenchu and Kurumba (P < 0.05). *Bifidobacterium longum* differed significantly between Chenchu and Irula (P < 0.05), and Irula and Konda Savara (P < 0.01), while *Blautia wexlerae* showed significance only between Chenchu and Konda Savara (P < 0.01) (Supplementary Fig. 11). Beyond population-based comparisons, we examined the impact of acculturation, sex, and alcohol consumption on the abundance of six disease-associated taxa irrespective of community. While no statistically significant differences were detected with these lifestyle-associated variables, we observed a consistent trend of increased abundance in more acculturated individuals, males compared to females, and alcohol drinkers compared to non-drinkers (Supplementary Fig. 12). These patterns appear to be influenced by lifestyle habits such as alcohol consumption, where females exhibited markedly lower drinking behaviour compared to males. In contrast, males displayed a significantly higher prevalence of alcohol use. The odds ratio (OR = 11.645; 95% CI: 5.362–25.291; P < 0.001) suggests that females were over 11 times more likely to be non-drinkers than males. This indicates that males are significantly more likely to consume alcohol in the studied population. Similarly, migration-associated dietary acculturation revealed sex differences, with females demonstrating significantly lower migration rates compared to males, who exhibited a markedly higher prevalence of occupational migration. The odds ratio (OR = 5.486; 95% CI: 2.272–13.248; P < 0.001) shows that females were over five times more likely to be non-migratory. These patterns suggest that sex-based differences in microbial abundance may reflect lifestyle-related behaviours rather than biological sex alone. To disentangle these effects, we conducted population-specific significance testing of disease-associated taxa across lifestyle variables, considering that not all taxa function as ‘candidaet keystone’ organisms in every community, based on their core association and structural importance in the gut microbiome (Supplementary Fig. 13). Notably, *Bifidobacterium longum* (R² = 0.16, P = 0.001, prevalence = 0.71) in Chenchu showed significant differences in acculturation (less acculturated [LA] vs. more acculturated [MA]) and smoking status. The MA group displayed an increasing abundance trend compared to the LA group. Conversely, higher abundance was observed among non-smokers than smokers, with an odds ratio of OR = 8.10 (95% CI: 1.456–45.062; P = 0.016), indicating that smoking was about eight times more prevalent in the LA group. This suggests that acculturation is a stronger driver of *B. longum* abundance than smoking behaviour alone (Fig. 9A and 9B). In contrast, *Bacteroides fragilis* (R² = 0.10, P = 0.001, prevalence = 0.51) in Chenchu showed significant differences across both acculturation and smoking habits, with increased abundance in the LA and smoker categories. The elevated prevalence of smokers in the LA group (∼8-fold higher) supports the inference that smoking behaviour contributes to the abundance of this taxon (Fig. 9C and 9D). Similarly, *Ruminococcus gnavus* (R² = 0.22, P = 0.002, prevalence = 0.70) in Jenu Kuruba showed a significant difference in acculturation status, with higher abundance in the MA group compared to the LA group (Fig. 9E). Finally, *Bacteroides fragilis* (R² = 0.12, P = 0.002, prevalence = 0.41) in Kurumba showed a significant difference by sex, with greater abundance in males (Fig. 9F). None of the females in the cohort reported smoking or drinking, while among the males, 4 of 18 reported smoking and 12 of 18 reported drinking. These results suggest that the observed sex-based differences in *B. fragilis* abundance may be indirectly driven by associated behaviours such as smoking and alcohol consumption, which are significantly more common among males.

## Discussion

The present study offers a comprehensive, high-resolution, ecosystem-based assessment of human gut microbial profiles across five culturally and ecologically distinct, particularly vulnerable tribal groups of India. By integrating taxonomic, functional, ecological, and lifestyle data, our findings indicate that traditional lifestyles and environmental exposures are significantly associated with gut microbial structure and concomitantly shape variation in health-associated microbial profiles. Metagenomic profiling seems to display both conserved as well as variable microbial sub-structures. Despite the dominance of common phyla such as *Bacteroidota* and *Firmicutes*, population-level variation was substantial.

At finer taxonomic scales, microbial core members like *Segatella spp*., *Faecalibacterium prausnitzii*, and *Prevotella sp. 885* were identified, which is consistent with rural and non-industrialized populations (Yatsunenko et al., 2012; Obregon-Tito et al., 2015). Intra-species divergence identified by SIMPER and LEfSe analyses, exemplified by the differential enrichment of *Segatella sinensis* in the Chenchus and *S. hominis* in the Kuruba and Konda Savara populations, is consistent with population-specific ecological associations and local environmental influences (Tett et al., 2019; Gupta et al., 2017).

Functionally, most populations retained a conserved core of essential metabolic pathways (e.g., glycolysis, folate transformation), but also exhibited population-specific enrichments. Xenobiotic degradation was prominent in Irula and Jenu Kuruba, likely reflecting with the exposure to phytochemically complex or environmentally distinct habitats, akin to other studies (Koppel et al., 2017). Chenchu showed amino acid biosynthesis enrichment, while Konda Savara was enriched for lipid metabolism, this observation is broadly consistent with previous reports of microbial metabolic responses associated with protein-limited, plant-based diets (Smits et al., 2017). Jenu Kuruba microbiome displayed increased mucin degradation and SCFA biosynthesis, which is consistent with potential host-microbe metabolic interactions (Schnorr et al., 2014; Rampelli et al., 2015).

Diversity metrics affirmed that traditional, comparatively less-acculturated groups (e.g., Chenchu, Jenu Kuruba) harbour richer microbial communities, aligning with prior findings (Yatsunenko et al., 2012; Schnorr et al., 2014; Rosas-Plaza et al., 2022).

Ordination analyses, including PCoA, dbRDA, and CCA, consistently demonstrated that population identity accounted for the greatest proportion of variation in gut microbial community composition among the metadata variables examined, outweighing individual behaviours (Rothschild et al., 2018; Charalampous et al., 2021). Interestingly, ecological overlap (e.g., Chenchu and Jenu Kuruba) yielded similar microbial signatures despite geographic separation, while subsistence similarity (e.g., Irula and Kurumba) did not ensure compositional resemblance, suggesting complex relationships among cultural, ecological, and geographic factors (Smits et al., 2017; Deschasaux et al., 2018).

Canonical Correspondence Analysis emphasized this interplay by showing strong vector alignment for Jenu Kuruba with *Alistipes onderdonkii*, associated with lipid metabolism and immune modulation (Kalnina et al., 2023). Irula, by contrast, showed minimal alignment, these findings are consistent with previous reports describing gut microbiome homogenization accompanying cultural transition (Rothschild et al., 2018; Gibbons et al., 2017).

The leave-one-taxon-out ordination framework identified 121 candidate keystone taxa, largely population-specific, with only a single taxon shared across all groups. This pattern suggests that the composition of candidate keystone taxa underpinning healthy microbial communities is context dependent rather than universally conserved. These findings are consistent with previous evidence demonstrating substantial inter-individual and population-level variation in the taxonomic composition of healthy gut microbiomes, which has made it difficult to define a universally conserved taxonomic profile of a healthy microbiome (Turnbaugh et al., 2007; Bäckhed et al., 2012; Lloyd-Price et al., 2016). While current study suggest that candidate keystone taxa associated with healthy microbial communities similarly vary across populations. Many candidate keystone taxa were low in abundance, yet showed strong associations with community structure, consistent with previous studies reporting important community-level associations of rare taxa (Jousset et al., 2017; Han et al., 2022). The repeated presence of *Oscillibacter spp*., *Phocaeicola vulgatus*, and *Alistipes putredinis* is consistent with the possibility of a context-dependent microbial core. To assess health relevance, we applied a comparative disease-association framework across 14 disease categories. Of the 121 candidate keystone taxa, 50 displayed consistent enrichment patterns, with 44 enriched in healthy individuals and six in disease cohorts, broadly consistent with previous microbiome meta-analyses (Tierney et al., 2019; Duvallet et al., 2017). Health-associated taxa, including *Fusicatenibacter saccharivorans, Ruminococcus lactaris*, and *Alistipes shahii*, are recognized producers of short-chain fatty acids and have been associated with anti-inflammatory functions (Mosca et al., 2016; Louis and Flint, 2016). Conversely, taxa such as *Bifidobacterium longum, Blautia wexlerae, Bacteroides fragilis*, and *Ruminococcus gnavus* were more frequently enriched in disease cohorts in our comparative analysis. These observations warrant cautious interpretation, as microbiome-disease associations are inherently context dependent and do not imply universal pathogenic or beneficial roles. *Bacteroides fragilis* and *Ruminococcus gnavus* have frequently been associated with inflammatory conditions and dysbiosis (Sears, 2009; Hall et al., 2017), whereas *Bifidobacterium longum* and *Blautia wexlerae* are widely recognized for their beneficial metabolic and immunomodulatory functions under many physiological conditions (O'Callaghan and van Sinderen, 2016; Liu et al., 2021; Valles-Colomer et al., 2019). Notably, *B. longum* exemplifies this context-dependent behaviour. Although traditionally regarded as a beneficial probiotic species, it was identified as disease-associated in our comparative analysis and has also been reported in conditions such as inflammatory bowel disease and schizophrenia. Current evidence suggests that the increased abundance of *B. longum* in these settings may represent a compensatory host response to oxidative stress and inflammation, potentially reflecting its antioxidant and immunomodulatory properties rather than an intrinsically pathogenic role (Zhao et al., 2021; Xu et al., 2022). Collectively, these observations underscore the dual roles of certain candidate keystone taxa and caution against assigning fixed beneficial or detrimental functions based solely on disease-association analyses.

Network analyses revealed taxa like *Flavonifractor plautii* (in Kurumba) and *B. wexlerae* (in Konda Savara) as central hubs. Their prominent associations with community structure suggest potential contributions to community stability while also indicating context-dependent associations with disease, aligning with previous observations of microbial community vulnerability and stability (Faust and Raes 2012; Banerjee et al., 2018). This reaffirms the need for network-informed therapeutic approaches (Shetty et al., 2016). Associations between candidate keystone taxa and lifestyle variables (sex, acculturation, alcohol, smoking) further illustrate microbiome context-dependence. Disease-associated taxa were more prevalent in acculturated individuals, males, and those with substance use, a trend consistent with earlier reports (Vangay et al., 2018; Shi et al., 2021; D’Aloisio et al., 2025).

Notably, women in this study had lower migration and substance use rates, raising the possibility that some observed sex-associated microbial differences are influenced by behavioural factors, although biological contributions cannot be excluded (Haro et al., 2016). In sum, our findings indicate that human gut microbial organization is not universal and is associated with a complex interplay of dietary, sociocultural, and population-specific factors. The candidate keystone taxa identified in this study were not necessarily the most abundant taxa but were computationally inferred to show stronger associations with community structure than other taxa. These findings support the value of culturally grounded, population-specific frameworks for microbiome research, particularly in underrepresented communities.

### Limitation

The present study is subject to several limitations. Its cross-sectional design and lack of longitudinal sampling preclude causal inference and assessment of the temporal stability of the identified candidate keystone taxa. Although the studied populations represent distinct traditional ecological settings, quantitative dietary intake was not systematically assessed, and the close interrelationship between geography, environment, and lifestyle may have contributed to the observed microbial variation. Comparative analyses relied predominantly on disease-associated metagenomic datasets from industrialized populations because of the limited availability of comparable datasets from traditional communities. Despite standardized reprocessing of all datasets, residual inter-study heterogeneity cannot be completely excluded, rendering the disease-association analyses exploratory. Finally, candidate keystone taxa were identified using an indirect computational inference framework based on community structural associations rather than experimental perturbation; therefore, their ecological roles require validation through longitudinal studies and complementary experimental approaches.

## Conclusion

This study provides the first high-resolution metagenomic characterization of the gut microbiome in five Particularly Vulnerable Tribal Groups (PVTGs) of Southern India, demonstrating how ecological settings, cultural practices, and lifestyle transitions shape microbial diversity, composition, and function. Integrative analyses reveal that gut microbiome organization is highly population-specific and strongly context-dependent. A total of 121 computationally inferred candidate keystone taxa were identified through their disproportionate contribution to community structure. These taxa exhibited pronounced population specificity with limited overlap among populations, challenging the concept of a universal core set of candidate keystone species. A subset of these taxa was consistently reproduced across geographically distinct traditional populations, suggesting that some candidate keystone taxa may represent conserved microbial features across traditional lifestyles while remaining strongly context-dependent. This observation generates hypotheses regarding their long-term persistence within gut microbial communities and warrants further investigation. Notably, several prioritized taxa were also associated with disease states, indicating that their contribution to community structure is not necessarily indicative of health-promoting functions. Multivariate ordination analyses identified population identity as a major correlate of gut microbial variation, likely reflecting the combined influence of ecological, cultural, and lifestyle differences among populations.

Less-acculturated groups exhibited higher microbial diversity, consistent with greater microbiome diversity and stability, whereas acculturation-related factors, including substance use and gender-linked practices, were associated with lower microbial diversity and greater enrichment of disease-linked taxa, highlighting associations between social and environmental factors and gut microbiome structure.

Overall, these findings highlight the value of a contextual framework for interpreting gut microbiome variation and emphasize the importance of culturally grounded, population-specific approaches in microbiome research involving underrepresented and transitioning communities.

## Declaration of generative AI and AI-assisted technologies in the manuscript preparation process

During the preparation of this work, the author(s) used ChatGPT and Grammarly to check grammar and language clarity. After using this tool/service, the author(s) reviewed and edited the content as needed and take(s) full responsibility for the content of the published article.

## Funding

The fieldwork and the research were funded by the Anthropological Survey of India (Ministry of Culture, Government of India) under its national project titled “Gut Microbial Genomic Study among the PVTGs of India”.

## Abbreviation

Candidate Keystone- Computationally inferred candidate keystone taxa are taxa whose abundance exhibits a disproportionately strong association with the multivariate organization of the residual microbial community following taxon removal and renormalization.

CCA- Canonical Correspondence Analysis

Core-associated taxa- Taxa with high explanatory contribution (R²), statistical significance (p < 0.05), and prevalence ≥65% across individuals.

CLR- Centered Log-Ratio dbRDA- Distance-based Redundancy Analysis

Envfit- Environmental Fitting Function

FDR- Benjamini–Hochberg False Discovery Rate

HS- Health-Association Score

Influential taxa-Taxa with high explanatory contribution (R²), statistical significance (p < 0.05), and moderate prevalence (30–64%) across individuals.

LA-Less acculturation

LDA- Linear Discriminant Analysis

LEfSe- Linear Discriminant Analysis Effect Size

MA-More acculturation

MetaCyc- Metabolic Pathway Database

PCoA-Principal Coordinate Analysis

PVTGs- Particularly Vulnerable Tribal Groups

SCFA- Short-chain fatty acid

SIMPER- Similarity Percentage Analysis

SN- Significantly Negative (Health-Associated)

SP- Significantly Positive (Disease-Associated)

TSS- Total Sum Scaling

## Credit Author Statement

Sahid Afrid Mollick (SAM) conceptualized and organized the study, performed bioinformatics and statistical analyses, and wrote the original draft. Gin Khan Khual (GKK) assisted in bioinformatics and statistical analyses. Ankita Ghosh (AG), Shiv Kumar Patel (SKP), Subhra Bhattacharyya (SB), and Chandra Sekhar Roy (CSR) assisted with statistical analyses. Hampapathalu Adimurthy Nagarajaram (HAN) and Anwesh Maile (AM) reviewed and edited the manuscript and input to the bioinformatics analysis. B.P. Urade (BPU) served as Principal Investigator of the study and was involved in manuscript review and editing; Mithun Sikdar (MS) served as Principal Investigator of the study and was involved in co-writing, manuscript review and editing; Moajungla Longkumer (ML), Mopada Nani Babu (MNB), Anup Raj Kundapur (ARK), Saloni Uniyal (SU), Arna Chatterjee (AC), and Maitrayee Mitra (MM) contributed to wet lab analyses, sample collection, and metadata preparation. Pulamaghatta N. Venugopal (PNV) conceptualized and supervised the study, performed bioinformatics and statistical analyses, and co-wrote the original draft.

## Declaration of competing interest

All authors declare that there is no conflict of interest regarding this article.

## Data Availability

The raw shotgun metagenomic sequencing data generated in this study are available in the European Nucleotide Archive (ENA) under BioProject accession PRJEB111164, with sample metadata provided in the Supplementary Tables. Publicly available datasets used for the health-association analyses, together with their accession details, are listed in the Resources File and Supplementary Table S3. The analysis code is available at link, and any additional information required to reproduce or reanalyse the study is available from the corresponding author upon reasonable request.

## Appendix Supplementary materials

Image, application 1

Image, application 2

Image, application 3

**Supplementary Fig. 1.** Represents comprehensive summary statistics of the sequencing depth statistics obtained from shotgun metagenomic sequencing.

**Supplementary Fig. 2.** Representation of the phylum-level relative abundance of the microbial taxa

**Supplementary Fig. 3.** Shared and population-specific microbial phyla across five tribal populations

**Supplementary Fig. 4.** Comparison of observed gut microbial diversity between Low Acculturation (LA) and Moderate Acculturation (MA) settlements across five tribal populations

**Supplementary Fig. 5.** distance-based redundancy (db-RDA) analysis of the microbial landscape with lifestyle-associated factors

**Supplementary Fig. 6.** Canonical Correspondence Analysis (CCA) ordination plot illustrating the influence of host-associated and lifestyle variables on gut microbial community structure

**Supplementary Fig. 7.** Represents the top most 20 variable metabolic pathways across the studied population.

**Supplementary Fig. 8.** Sensitivity analysis of prevalence and R² thresholds used for candidate keystone taxon identification. (A) Mean cumulative abundance retained across prevalence thresholds. (B) Mean pairwise Jaccard similarity among population-specific core taxa. (C–D) Sample-level distributions of cumulative abundance and Jaccard similarity at the 30% and 65% prevalence thresholds, respectively. The selected prevalence thresholds represent a balance between microbial community representation and inter-population reproducibility. (E) Empirical distribution of R² values across all taxon–population observations. The red dashed line indicates the empirical 65th percentile (R² = 0.018), evaluated as a data-driven threshold for high association strength. Taxa exceeding this threshold and satisfying P < 0.05 were classified as core-associated (prevalence ≥65%) or influential (prevalence 30–64%).

**Supplementary Fig. 9.** Faceted volcano plots showing pairwise differential abundance of microbial species across tribal populations

**Supplementary Fig. 10.** Population-specific and integrated microbial co-occurrence network centrality analysis showing the top 15 taxa ranked by degree, betweenness, and closeness centrality. Disease-associated taxa are highlighted in red.

**Supplementary Fig. 11.** Comparative relative abundance of six disease-associated bacterial taxa across five tribal populations

**Supplementary Fig. 12.** Population-stratified mean relative abundances of six disease-associated microbial taxa across lifestyle-associated variables, including acculturation level, chewing habit, sex, alcohol consumption, and smoking status

**Supplementary Fig. 13.** Population-specific distribution of disease-associated candidate keystone taxa depicting their association with community structure (R²) and prevalence.

**Supplementary Fig. 14.** Cumulative contribution plot of gut-associated 489 microbial taxa based on SIMPER analysis, illustrating the ranked dissimilarity contributions of 1,542 identified taxa across studied populations

**Supplementary Fig. 15.** Cross-cohort reproducibility of core candidate keystone taxa identified in the studied tribal populations using an independent, geographically distinct tribal validation cohort (Panel A), corresponding health-association profiles (Panel B), and cross-cohort concordance of stability scores between the Indian discovery and African validation cohorts (Panel C).

**Supplementary Table S1.** Metadata information with sample ID.

**Supplementary Table S2.** Inclusion and exclusion criteria for the study cohort.

**Supplementary Table S3.** Secondary source–derived metadata and sample information.

**Supplementary Table S4.** Broad classification of retrieved data by disease category.

**Supplementary Table S5.** Summarization of classified disease cohort.

**Supplementary Table S6.** 10 iteration-based health-association score (HS).

**Supplementary Table S7.** Cross-cohort validation using independent traditional gut metagenomes.

**Supplementary Table S8.** Landscape of microbial relative abundance and prevalence among the studied cohort.

**Supplementary Table S9.** Functional Landscape of microbial relative abundance among the studied cohort.

**Supplementary Table S10.** Prevalence of the candidate keystone taxa across the studied population.

**Supplementary Table S11.** Cross-cohort reproducibility of core candidate keystone taxa.

**Supplementary Table S12.** Health-association of reproducible core candidate keystone taxa.

**Supplementary Table S13.** Formal expression of candidate keystone taxa identification.
