# Supplementary material for: Gut microbial diversity and candidate keystone taxa in Indian tribes: Insights across lifestyle-ecological continuum and health associations
Source: Curr Res Microb Sci. 2026 Jul 24;11:100650. doi: 10.1016/j.crmicr.2026.100650 (PMC13470565; doi:10.1016/j.crmicr.2026.100650)
Supplement: Supplementary file 1 [file mmc2.docx]

**Gut Microbial Diversity and Candidate Keystone Taxa in Indian Tribes: Insights Across Lifestyle-ecological continuum and Health Associations**

**Resource**

**Key resources to execute the present study**

| **Experimental Material**  **(Reagents and software)** | **Source** | **Reference** |
| --- | --- | --- |
| **Wet-lab analysis (Deta generation)** | | |
| OMNIgene Gut; OMR-200; Faecal matter collection tube for Microbial DNA | DNA Genotek Inc. (Canada) | https://www.dnagenotek.com/us/pdf/PD-BR-00159.pdf |
| QIAamp PowerFecal Pro DNA Kit  Cat no. / ID.   51804 | Qiagen (Germany) | https://www.qiagen.com/us/products/discovery-and-translational-research/dna-rna-purification/dna-purification/genomic-dna/qiaamp-powerfecal-pro-dna-kit |
| **Dry-lab analysis (Data quartino and analysis)** | | |
| Analysed Data | This Study |  |
| Publicly Available Data | CuratedMetagenomicData accession numbers are listed in the Supplementary Table 4 | https://doi.org/10.1038/nmeth.4468 |
| Human genome (hg38) | NCBI | https://www.ncbi.nlm.nih.gov/datasets/genome/GCF_000001405.26/ |
| **Software and Analysis Code** | | |
| FastQC v0.12.1 | Andrews, 2010 | https://www.bioinformatics.babraham.ac.uk/projects/fastqc/ |
| MultiQC | Ewels et al., 2016 | https://doi.org/10.1093/bioinformatics/btw354 |
| BBDuk | Bushnell, 2014 | https://sourceforge.net/projects/bbmap/ |
| Fastp | Chen et al., 2018 | https://doi.org/10.1093/bioinformatics/bty560 |
| Kraken 2 | Wood et al., 2019 | https://doi.org/10.1186/s13059-019-1891-0 |
| Hostile | Constantinides et al., 2023 | https://doi.org/10.1093/bioinformatics/btad728 |
| MetaPhlAn v4.1 | Blanco-Míguez et al., 2023 | https://doi.org/10.1038/s41587-023-01688-w |
| HumanN v3.9 | Franzosa et al., 2018 | https://github.com/biobakery/humann |
| MetaCyc | Caspi et al., 2019 | https://doi.org/10.1093/nar/gkz862 |
| R version 4.5.0 | R Core Team (2025) | https://www.r-project.org/ |
| vegan | Oksanen et al., 2025 | <https://vegandevs.github.io/vegan/> |
| ggplot2 | Wickham et al.,2025 | https://doi.org/[10.32614/CRAN.package.ggplot2](https://doi.org/10.32614/CRAN.package.ggplot2) |
| patchwork | Pedersen et al.,2025 | [https://patchwork.data-imaginist.com](https://patchwork.data-imaginist.com/) |
| grid | Paul Murrell, 2025 | https://stat.ethz.ch/R-manual/R-devel/library/grid/doc/grid.pdf |
| dplyr | Muller et al., 2018 | https://www.tidyverse.org/blog/2018/06/dplyr-0.7.5/ |
| ggrepel | Kamil Slowikowski, 2024 | https://cran.r-project.org/web/packages/ggrepel/vignettes/ggrepel.html |
| tidyr | Wickham et al., 2024 | https://doi.org/[10.32614/CRAN.package.tidyr](https://doi.org/10.32614/CRAN.package.tidyr) |
| ggraph | Pedersen T, 2024 | [https://ggraph.data-imaginist.com](https://ggraph.data-imaginist.com/) |
| igraph | Csardi & Nepusz, 2006 | https://github.com/igraph/igraph |
| tidyverse | Wickham et al., 2019 | https://[doi.org/10.21105/joss.01686](https://doi.org/10.21105/joss.01686) |
| reshape2 | Wickham, 2007 | https://doi.org/[10.18637/jss.v021.i12](https://doi.org/10.18637/jss.v021.i12) |
| Hmisc | Harrell et al., 2019 | https://cran.uib.no/web/packages/Hmisc/Hmisc.pdf |
| RColorBrewer | Erich Neuwirth, 2022 | https://cran.r-project.org/web/packages/RColorBrewer/RColorBrewer.pdf |
| compositions | K. Gerald van den Boogaart & R. Tolosana-Delgado, 2008 | <https://doi.org/10.1016/j.cageo.2006.11.017> |
| tidygraph | Thomas Lin Pedersen, 2024 | https://cran.r-project.org/web/packages/tidygraph/tidygraph.pdf |
| ggalluvial | Brunson et al, 2018 | https://ftp2.uib.no/cran/web/packages/ggalluvial/ggalluvial.pdf |
| ggpubr | Kassambara A, 2025 | <https://rpkgs.datanovia.com/ggpubr/> |
| pheatmap | Kolde, Raivo & Maintainer Raivo Kolde, 2015 | https://cran.ms.unimelb.edu.au/web/packages/pheatmap/pheatmap.pdf |
| VennDiagram | Hanbo Chen, 2022 | https://cran.r-project.org/web/packages/VennDiagram/VennDiagram.pdf |
| gridExtra | Baptiste Auguie & Anton Antonov, 2025 | https://github.com/cran/gridExtra |
| UpSetR | Conway et al, 2017 | https://doi.org/[10.1093/bioinformatics/btx364](https://doi.org/10.1093/bioinformatics/btx364) |
| readr | Wickham H, Hester J, Bryan J, 2024 | [https://readr.tidyverse.org](https://readr.tidyverse.org/) |
| ExperimentHub | Morgan M & Shepherd L, 2025 | https://doi.org/[10.18129/B9.bioc.ExperimentHub](https://doi.org/doi:10.18129/B9.bioc.ExperimentHub) |
| SummarizedExperiment | Morgan et al., 2025 | https://doi.org/[10.18129/B9.bioc.SummarizedExperiment](https://doi.org/doi:10.18129/B9.bioc.SummarizedExperiment) |
| qvalue | Storey, 2025 | https://doi.org/[10.18129/B9.bioc.qvalue](https://doi.org/doi:10.18129/B9.bioc.qvalue) |
| stats | R Core Team | https://stat.ethz.ch/R-manual/R-devel/library/stats/html/stats-package.html |
| data.table | Barrett et al, 2025 | [https://r-datatable.com](https://r-datatable.com/) |
| ComplexHeatmap | Zuguang Gu, 2016 | https://doi.org/[10.18129/B9.bioc.ComplexHeatmap](https://doi.org/doi:10.18129/B9.bioc.ComplexHeatmap) |
| circlize | Zuguang Gu at al, 2014 | <https://doi.org/10.1093/bioinformatics/btu393> |
| LEfse | Asya Khleborodova, 2024 | <https://doi.org/10.1093/bioinformatics/btae707> |
| DEseq2 | Love et al, 2014 | https://doi.org/[10.18129/B9.bioc.DESeq2](https://doi.org/doi:10.18129/B9.bioc.DESeq2) |
| Analysis codes are deposited | GitHub | https://github.com/Sahid96/Keystone_taxa_and_health_associations.git |
