# Supplementary material for: Gut microbial diversity and candidate keystone taxa in Indian tribes: Insights across lifestyle-ecological continuum and health associations
Source: Curr Res Microb Sci. 2026 Jul 24;11:100650. doi: 10.1016/j.crmicr.2026.100650 (PMC13470565; doi:10.1016/j.crmicr.2026.100650)
Supplement: Supplementary file 2 [file mmc3.docx]

**Gut Microbial Diversity and Candidate Keystone Taxa in Indian Tribes: Insights Across Lifestyle-Ecological Continuum and Health Associations**

**Supplementary Figures and Tables**

**Supplementary Figure 1: Represents comprehensive summary statistics of the sequencing depth statistics obtained from shotgun metagenomic sequencing**

A total of 182 fecal samples were collected from five Particularly Vulnerable Tribal Groups (PVTGs). Sequencing depth per sample ranged from ~13 million to >41 million reads, yielding ~4.46 billion reads in total. The mean read depth was ~24.5 million reads per sample, with a small standard error, indicating consistent sequencing performance across samples. Variability in sequencing depth was moderate (CV ~16.3%), which is acceptable for large-scale shotgun metagenomic studies. The median read depth (24.2 million) closely matched the mean, suggesting an approximately symmetric distribution with a slight right skew, driven by a few higher-depth samples. Most samples clustered within a narrow interquartile range (21.6–27.0 million reads), reflecting well-balanced coverage. Overall, the sequencing depth was sufficient and uniform across samples, supporting robust downstream taxonomic and functional analyses of the gut microbiome.

**Supplementary Figure 2: Representation of the phylum-level relative abundance of the microbial taxa.**

This stacked bar plot represents the relative abundance of gut microbial phyla across five tribal populations: Irula, Jenu Kuruba, Kurumba, Chenchu, and Konda Savara. Each bar represents a single population, and the segments within each bar indicate the proportional contribution of individual phyla to the overall gut microbiota composition, expressed as a percentage. The color-coded legend on the right identifies the phyla contributing to each population's microbial profile. The visualization reveals both conserved and variable components of the gut microbiota at the phylum level across the studied groups.

Across all populations, *Bacteroidota* and *Firmicutes* are the two most dominant phyla, together accounting for more than 85% of the total microbial abundance in each group. Irula displays the highest relative abundance of *Bacteroidota* (63.28%), followed by Chenchu (59.88%), Jenu Kuruba (58.51%), Kurumba (57.19%), and Konda Savara (53.86%). Conversely, *Firmicutes* ranges from 27.59% in Irula to 35.45% in Konda Savara, showing a reciprocal relationship with *Bacteroidota*. This pattern indicates that although both phyla are dominant, their proportions vary in a population-specific manner, potentially reflecting dietary fiber intake or carbohydrate fermentation profiles unique to each community.

Proteobacteria is the third most abundant phylum in all five populations, with values ranging between 3.66% (Irula) and 6.59% (Konda Savara), while *Candidatus_Melainabacteria*, *Actinobacteria*, and *Tenericutes* collectively contribute to a smaller but consistent proportion of the community. Notably, the presence of *Verrucomicrobia*, *Elusimicrobia*, and *Lentisphaerae* further reflects the presence of more specialized or less abundant taxa. *Synergistetes* and *Candidatus_Thermoplasmatota* are present at very low levels or absent in some groups, suggesting sporadic distribution or environmental variability in their colonization.

A key distinction arises in the presence of rare or population-specific phyla. *Fornicata* is present only in Irula and Konda Savara, whereas Ascomycota appears only in Chenchu and Konda Savara, suggesting potential environmental or dietary sources specific to these groups. *Candidatus_Saccharibacteria* is uniquely found in Chenchu, and *Evosea* is exclusive to Konda Savara, albeit in minimal abundance. These low-abundance but distinct taxa contribute to the microbial individuality of each population and may serve as biomarkers of unique ecological or cultural traits.

Altogether, the plot highlights a strong microbial core comprising *Bacteroidota*, *Firmicutes*, and Proteobacteria across all groups, while also exposing subtle but meaningful variation in the presence and abundance of less dominant phyla. These inter-population differences may reflect underlying lifestyle, dietary, or environmental differences among the tribal communities.

**Supplementary Figure 3: Shared and population-specific microbial phyla across five tribal populations.**

This figure shows the distribution and overlaps of microbial phyla across five tribal populations based on presence–absence data. The horizontal bars indicate the total number of phyla detected in each group, with all populations showing the same richness (15 phyla), suggesting comparable phylum-level diversity. The UpSet plot highlights shared and unique phyla, where the dot matrix denotes the populations involved in each intersection and the bar heights represent the number of phyla common to those groups. The tallest bar on the far right, with a count of 13, corresponds to the 13 microbial phyla that are common across all five populations, indicating a core set of gut microbial phyla widely shared irrespective of lifestyle, geography, or ethnic differences. These commonly shared phyla include *Bacteroidota*, *Firmicutes,* *Proteobacteria*, *Candidatus_Melainabacteria*, *Actinobacteria*, *Tenericutes*, *Fusobacteria*, *Lentisphaerae*, *Elusimicrobia*, *Verrucomicrobia*, *Spirochaetes*, *Synergistetes*, and *Euryarchaeota*. The consistent presence of these phyla across groups highlights a functionally conserved microbial core likely essential for maintaining fundamental gut functions.

The smaller bars on the left, each with a value of 1, represent phyla that are uniquely shared by one or two populations. For instance, *Fornicata* is found only in Irula, *Candidatus_Saccharibacteria* appears uniquely in Chenchu, and *Evosea* is exclusive to Konda Savara. Ascomycota is detected only in Chenchu and Konda Savara, while *Candidatus_Thermoplasmatota* is exclusive to Kurumba. These unique phyla may reflect population-specific environmental exposures, dietary practices, or cultural habits influencing the microbiome composition.

Overall, the figure conveys that while there is a strong core microbiome common to all groups at the phylum level, subtle differences exist that are unique to individual or pairs of populations. These distinctions may have implications for understanding local adaptations, microbiome-driven health outcomes, and ecological variation among these tribal communities.

**Supplementary Figure 4: Comparison of observed gut microbial diversity between Low Acculturation (LA) and Moderate Acculturation (MA) settlements across five tribal populations.**

| Population | W | P | Median_LA | Median_MA | Effect_Size | Magnitude | FDR-adjusted P | Significance |
| --- | --- | --- | --- | --- | --- | --- | --- | --- |
| Chenchu | 279 | 0.018887 | 228 | 173.5 | 0.400470466 | moderate | 0.047216578 | * |
| Irula | 131 | 0.181701 | 204 | 179 | 0.161109424 | small | 0.227126224 | ns |
| Jenu Kuruba | 71 | 0.000283 | 229.5 | 193 | 0.342274956 | moderate | 0.001415725 | ** |
| Konda Savara | 255.5 | 0.162761 | 242 | 215 | 0.23983415 | small | 0.227126224 | ns |
| Kurumba | 195.5 | 0.298355 | 229 | 204 | 0.179597391 | small | 0.298354862 | ns |

*Within-population comparison of observed gut microbial richness between acculturation groups, including Wilcoxon rank-sum test statistics, effect sizes, and FDR-adjusted *P*-values.

Boxplots showing observed microbial richness across the five tribal populations stratified by acculturation status, with less acculturated (LA) and more acculturated (MA) settlements represented separately. Observed richness was quantified as the number of detected microbial taxa per sample. Chenchu individuals from LA settlements exhibited significantly higher microbial richness than those from MA settlements (median = 228 vs. 173.5 taxa; Wilcoxon rank-sum test: raw *P* = 0.0189, FDR-adjusted *P* = 0.0472; effect size *r* = 0.40, moderate). Conversely, Jenu Kuruba individuals from MA settlements displayed significantly greater richness than those from LA settlements (median = 229.5 vs. 193 taxa; raw *P* = 2.83 × 10⁻⁴, FDR-adjusted *P* = 0.0014; *r* = 0.34, moderate). No significant differences were observed between LA and MA groups in the Irula (raw *P* = 0.182, FDR-adjusted *P* = 0.227; *r* = 0.16), Konda Savara (raw *P* = 0.163, FDR-adjusted *P* = 0.227; *r* = 0.24), or Kurumba (raw *P* = 0.298, FDR-adjusted *P* = 0.298; *r* = 0.18) populations, all of which exhibited small effect sizes. Overall, these findings indicate that the influence of acculturation on gut microbial richness is population-specific, with moderate effects observed only in Chenchu and Jenu Kuruba, whereas the remaining populations showed comparatively small differences despite variation in median richness.

**Supplementary Figure 5: distance-based redundancy (db-RDA) analysis of the microbial landscape with lifestyle-associated factors.**

This bar plot summarizes the proportion of variation in microbial composition that can be explained by six host-related factors: population, sex, acculturation, drinking, chewing, and smoking. The x-axis denotes the percentage of variance explained, and each blue bar represents a variable’s contribution to the total variation, with associated error bars showing standard error. Asterisks denote statistical significance levels.

Among all variables, population explains the largest proportion of microbial variation (~3.07%) with high statistical significance (P = 0.001), highlighting its dominant role in shaping gut microbiota. Sex and acculturation are the next most influential variables, explaining ~0.76% and ~0.66% of the variance respectively, both statistically significant (P = 0.003 and P = 0.005), indicating that gender differences and degree of acculturation also play important roles in microbial community structure. On the other hand, lifestyle habits such as drinking, chewing, and smoking explain much smaller proportions of variation (each less than 0.2%) and are not statistically significant (P> 0.05), suggesting these behaviours contribute little to inter-individual microbiota differences within this cohort.

The findings underscore that cultural identity, biological sex, and integration into modern lifestyles are stronger determinants of microbial diversity in these tribal populations compared to substance-use behaviours.

**Supplementary Figure 6: Canonical Correspondence Analysis (CCA) ordination plot illustrating the influence of host-associated and lifestyle variables on gut microbial community structure.**

The Canonical Correspondence Analysis (CCA) model output summarizes how environmental or host-associated variables explain variation in microbial community structure. The model shows that the overall constrained ordination is statistically significant (Chi-square = 0.216, *F* = 3.96, *p* = 0.001), indicating that the explanatory variables collectively account for a meaningful portion of the community variation. The first five axes (CCA1 to CCA5) are individually significant, with Axis 1 and 2 explaining 5.3% and 3.88% of the variance, respectively, summing to a cumulative 9.18%. This suggests that while most of the variation remains unexplained (typical in ecological datasets), the constrained portion still reveals statistically robust ecological gradients influenced by key factors like population, sex, and acculturation. Subsequent axes (CCA6 onwards) contribute minimally and are not statistically significant, implying diminishing explanatory power.

**Supplementary Figure 7: Represents the top most 20 variable metabolic pathways across the studied population.**

The heatmap represents the twenty key metabolic pathways across five distinct tribal communities: Irula, Jenu Kuruba, Kurumba, Chenchu, and Konda Savara. These twenty pathways were selected based on their highest variance across the populations, indicating their potential relevance in capturing functional differences in the gut microbiome between the groups. Each pathway contributes to core microbial functions and reflects potential metabolic specialization or environmental and dietary adaptation in different communities.

One of the most prominent pathways is the "dTDP-β-L-rhamnose biosynthesis" pathway, consistently showing the highest abundance across all five communities. This pathway is crucial for the biosynthesis of rhamnose, a sugar component of bacterial cell walls and lipopolysaccharides, implicating it in bacterial structural integrity and host-microbiome interaction. The high abundance of this pathway in the Irula and Chenchu groups likely reflects a greater presence of rhamnose-producing microbes in these communities. Another pathway showing marked variability is queuosine biosynthesis I (de novo). Queuosine is a modified building block of certain tRNAs and plays an important role in maintaining accurate protein synthesis. Its increased abundance in the Chenchu and Konda Savara populations suggests that the gut microbes in these communities may be better equipped with efficient protein-producing systems. By improving the accuracy of protein synthesis and reducing the production of faulty or energetically costly proteins, this enhanced translational capacity may help microbes function more efficiently, cope with environmental stresses, and maintain stable colonization, ultimately contributing to improved microbial fitness in these distinct gut environments.

Pathways such as "L-isoleucine biosynthesis III" and "L-isoleucine biosynthesis I (from threonine)" represent alternative microbial biosynthetic routes for the essential amino acid isoleucine. Their simultaneous presence and variability highlight microbial plasticity in amino acid synthesis, possibly driven by differences in host diet or microbial community composition. Notably, the Jenu Kuruba and Kurumba populations show a higher abundance of these amino acid biosynthetic pathways.

Energy metabolism is represented through pathways like "pyruvate fermentation to isobutanol (engineered)", "glycolysis IV", and "glucose and glucose-1-phosphate degradation". These pathways relate to central carbon metabolism and indicate how microbes process carbohydrates. "Glycolysis IV" and glucose degradation are particularly enriched in the Irula and Kurumba groups, suggesting more efficient microbial carbohydrate metabolism in these populations, possibly linked to higher dietary fiber intake.

Nucleotide metabolism is another major theme among the top variable pathways. Several superpathways such as "guanosine ribonucleotides de novo biosynthesis", "superpathway of guanosine nucleotides de novo biosynthesis I", "superpathway of adenosine nucleotides de novo biosynthesis I and II", and "superpathway of pyrimidine nucleobases salvage", are abundant and show differential representation across groups. These pathways suggest active microbial DNA and RNA synthesis, consistent with fast-growing bacterial populations. Their enrichment in the Irula and Jenu Kuruba communities may indicate greater microbial turnover or transcriptional activity.

Other pathways such as the "methylerythritol phosphate pathway II", involved in isoprenoid biosynthesis, and the "superpathway of branched chain amino acid biosynthesis", critical for synthesizing essential amino acids like leucine, isoleucine, and valine, point toward important microbial contributions to host nutrition. The relatively higher abundance of these pathways in Kurumba and Konda Savara communities could be linked to microbial strategies for adapting to nutrient-limited environments.

The "glycogen biosynthesis I (from ADP-D-glucose)" and "peptidoglycan biosynthesis I (meso-diaminopimelate containing)" pathways highlight microbial storage and cell wall synthesis capabilities, respectively. Their consistent representation across all communities underscores their essential roles in microbial survival and colonization of the gut.

The heatmap visually summarizes the same data, presenting normalized abundance values for each pathway across the five populations. It uses a color gradient to indicate relative abundance, where deeper shades represent higher expression levels. The pathways are arranged in order based on their variability, making it easier to identify functionally distinct microbial signatures across populations. For example, pathways such as dTDP-rhamnose biosynthesis and nucleotide biosynthesis appear in darker hues across all communities, especially in Irula and Chenchu, suggesting their core presence and possible overrepresentation in these groups. In contrast, pathways like glycogen biosynthesis or pyruvate fermentation to isobutanol show more variability, possibly representing community-specific microbial functions.

Overall, the table and heatmap together highlight population-specific microbial functional profiles shaped by ecological, dietary, and cultural influences. They provide insight into how microbial communities adapt their metabolic capacities in response to differing lifestyles and environments of Indian tribal populations, and offer a window into the role of the gut microbiome in human adaptation.

**Supplementary Figure 8. Sensitivity analysis of prevalence and R² thresholds used for candidate keystone taxon identification. (A) Mean cumulative abundance retained across prevalence thresholds. (B) Mean pairwise Jaccard similarity among population-specific core taxa. (C–D) Sample-level distributions of cumulative abundance and Jaccard similarity at the 30% and 65% prevalence thresholds, respectively. The selected prevalence thresholds represent a balance between microbial community representation and inter-population reproducibility. (E) Empirical distribution of R² values across all taxon–population observations. The red dashed line indicates the empirical 65th percentile (R² = 0.018), evaluated as a data-driven threshold for high association strength. Taxa exceeding this threshold and satisfying P < 0.05 were classified as core-associated (prevalence ≥65%) or influential (prevalence 30–64%).**

| Threshold | Mean_Jaccard | SD_Jaccard | Mean_Abundance | SD_Abundance | Retained_Taxa |
| --- | --- | --- | --- | --- | --- |
| 0.05 | 0.702505 | 0.032309 | 99.08061 | 2.178088 | 422 |
| 0.1 | 0.702505 | 0.032309 | 97.59075 | 4.495769 | 365 |
| 0.15 | 0.702505 | 0.032309 | 97.13148 | 4.654618 | 330 |
| 0.2 | 0.702505 | 0.032309 | 94.5746 | 6.309808 | 298 |
| 0.25 | 0.716865 | 0.025582 | 92.90345 | 7.630158 | 272 |
| 0.3 | 0.704707 | 0.04731 | 91.54488 | 8.892426 | 242 |
| 0.35 | 0.699104 | 0.046446 | 89.35603 | 9.666848 | 223 |
| 0.4 | 0.644086 | 0.048714 | 87.59998 | 10.13712 | 201 |
| 0.45 | 0.633765 | 0.052911 | 81.95057 | 11.34627 | 173 |
| 0.5 | 0.619954 | 0.056467 | 79.03062 | 12.12889 | 140 |
| 0.55 | 0.627054 | 0.060168 | 76.04055 | 12.69917 | 118 |
| 0.6 | 0.635236 | 0.045163 | 69.50396 | 14.1527 | 102 |
| 0.65 | 0.630211 | 0.060413 | 64.88353 | 14.6036 | 85 |
| 0.7 | 0.638665 | 0.062102 | 57.55684 | 14.61277 | 73 |
| 0.75 | 0.607289 | 0.065541 | 47.05818 | 14.8784 | 60 |
| 0.8 | 0.599539 | 0.062363 | 39.38756 | 15.64622 | 47 |
| 0.85 | 0.616831 | 0.093397 | 36.95468 | 16.12866 | 37 |
| 0.9 | 0.499497 | 0.126406 | 36.46168 | 16.19103 | 30 |
| 0.95 | 0.327894 | 0.192835 | 10.5966 | 5.753074 | 16 |

*Sensitivity analysis for prevalence threshold selection

| Percentile | R2_Cutoff | Number_Selected | Median_R2 | Median_Pvalue | Mean_Pvalue | Median_Prevalence | Mean_Prevalence | Percent_P_less_0.05 | Percent_Core | Percent_Influential | Percent_LowPrev |
| --- | --- | --- | --- | --- | --- | --- | --- | --- | --- | --- | --- |
| 50 | 0.010015 | 868 | 0.033733 | 0.039 | 0.052949 | 0.451613 | 0.500416 | 56.8 | 26.38 | 48.39 | 25.23 |
| 55 | 0.012495 | 781 | 0.038684 | 0.031 | 0.043046 | 0.472222 | 0.507508 | 63.12 | 27.14 | 49.17 | 23.69 |
| 60 | 0.015961 | 694 | 0.044933 | 0.0235 | 0.034689 | 0.472222 | 0.509925 | 71.04 | 27.38 | 49.57 | 23.05 |
| 65 | 0.019902 | 607 | 0.052135 | 0.018 | 0.027405 | 0.472222 | 0.512547 | 80.56 | 27.68 | 49.75 | 22.57 |
| 70 | 0.025737 | 521 | 0.061086 | 0.014 | 0.021088 | 0.483871 | 0.517503 | 88.48 | 28.79 | 47.98 | 23.22 |
| 75 | 0.033733 | 434 | 0.070683 | 0.009 | 0.015164 | 0.483871 | 0.522046 | 95.62 | 29.26 | 48.39 | 22.35 |
| 80 | 0.044901 | 347 | 0.084549 | 0.006 | 0.010905 | 0.487179 | 0.52539 | 97.98 | 29.68 | 48.41 | 21.9 |
| 85 | 0.061084 | 261 | 0.104098 | 0.004 | 0.007456 | 0.5 | 0.52983 | 98.85 | 30.65 | 48.28 | 21.07 |
| 90 | 0.084174 | 174 | 0.133343 | 0.002 | 0.003759 | 0.520565 | 0.551482 | 100 | 35.06 | 44.25 | 20.69 |

* Evaluation of empirical R² thresholds for candidate keystone taxon identification

| Percentile | R2_Cutoff | Number_Selected | Median_R2 | Median_Prevalence | Mean_Prevalence | Core_Associated | Influential | Low_Prevalence |
| --- | --- | --- | --- | --- | --- | --- | --- | --- |
| 50 | 0.064069 | 247 | 0.108413 | 0.512821 | 0.534861 | 78 | 118 | 51 |
| 55 | 0.069817 | 222 | 0.11681 | 0.516129 | 0.536432 | 69 | 108 | 45 |
| 60 | 0.07692 | 197 | 0.127291 | 0.525 | 0.545329 | 65 | 92 | 40 |
| 65 | 0.084619 | 173 | 0.133714 | 0.525 | 0.552991 | 61 | 77 | 35 |
| 70 | 0.09548 | 148 | 0.143562 | 0.525 | 0.563607 | 55 | 66 | 27 |
| 75 | 0.108413 | 124 | 0.157344 | 0.538462 | 0.565043 | 47 | 56 | 21 |
| 80 | 0.127178 | 99 | 0.16609 | 0.575 | 0.582281 | 42 | 38 | 19 |
| 85 | 0.143216 | 74 | 0.197341 | 0.586538 | 0.595855 | 33 | 28 | 13 |
| 90 | 0.165979 | 50 | 0.223106 | 0.67621 | 0.62281 | 26 | 15 | 9 |

* Evaluation of significant candidate keystone taxa across empirical R² thresholds

To determine whether the prevalence thresholds used to define core-associated (≥65%) and influential (≥30%) taxa were empirically justified rather than arbitrarily selected, we performed a prevalence sensitivity analysis across thresholds ranging from 5% to 95% in 5% increments. At each threshold, taxa satisfying the specified prevalence criterion within each tribal population were identified, and pairwise Jaccard similarity coefficients were calculated to quantify the reproducibility of the resulting taxon sets across populations. In parallel, we quantified the cumulative community abundance and the number of retained taxa to assess the balance between community representation and feature retention. An objective knee-point analysis of the cumulative abundance curve was then used to identify the transition from permissive to increasingly stringent prevalence filtering.

As shown in Supplementary Figure 8A, progressively increasing the prevalence threshold reduced both the number of retained taxa and the cumulative community abundance. Although community abundance declined gradually across lower and intermediate thresholds, a clear inflection was observed at approximately 55% prevalence, beyond which increasingly stringent filtering resulted in disproportionately greater losses of community representation. A similar pattern was observed for reproducibility, where mean pairwise Jaccard similarity remained relatively stable between approximately 55% and 70% prevalence (Supplementary Figure 8B), indicating that stricter filtering within this interval had minimal impact on the consistency of retained taxa across populations. Beyond this range, cumulative abundance declined sharply while improvements in reproducibility were marginal, suggesting diminishing returns from further increasing the prevalence requirement.

The complementary sample-level sensitivity analyses (Supplementary Figure 8C–D) further supported the selected prevalence thresholds. A 30% prevalence threshold retained a substantially larger fraction of the microbial community while excluding only infrequently detected taxa, thereby providing sufficient coverage to identify taxa with potential structural influence despite moderate prevalence. Conversely, the 65% threshold fell within the region of stable inter-population reproducibility while preserving most community representation, providing a conservative criterion for defining taxa consistently detected across the majority of individuals. Collectively, these findings indicate that the prevalence thresholds used to define influential and core-associated taxa represent complementary, data-driven trade-offs between community coverage, feature retention, and reproducibility.

To objectively evaluate the definition of high association strength, we additionally examined the empirical distribution of R² values obtained from the leave-one-taxon-out *envfit* analysis. The empirical 65th percentile of the R² distribution (R² = 0.0199) was evaluated as a data-driven reference threshold for strong structural association. Sensitivity analysis across R² percentiles (50th–90th) demonstrated progressive enrichment for statistically significant associations, with the proportion of taxa satisfying P < 0.05 increasing from 56.8% at the 50th percentile to 100% at the 90th percentile, while the number of retained taxon–population observations decreased from 868 to 174. The 65th percentile represented a balanced compromise, retaining 607 observations while enriching the retained set to 80.6% statistically significant associations. Among statistically significant taxa, increasing the R² threshold progressively reduced the numbers of both core-associated and influential taxa but preserved both prevalence-defined categories across all evaluated thresholds. Together, these analyses demonstrate that the prevalence and R² thresholds used in this study were empirically supported rather than arbitrarily selected, providing an objective basis for identifying candidate keystone taxa.

**Supplementary Figure 9: Faceted volcano plots showing pairwise differential abundance of microbial species across tribal populations.**

Each panel represents a pairwise comparison between two tribal populations. The x-axis denotes log<sub>2</sub> fold change (log2FC) in species abundance, and the y-axis indicates the -log<sub>10</sub> of the adjusted p-value (padj) derived from DESeq2 analysis. Species that showed significant differential abundance (|log<sub>2</sub>FC| > 2 and padj < 0.05) are highlighted in red, while non-significant taxa are shown in grey. Dashed vertical lines mark the ±2 log<sub>2</sub> fold change threshold, and the horizontal dashed line corresponds to the multiple testing-corrected significance level of 0.05. The plot provides a comparative overview of species that are enriched or depleted between populations.

This figure is essential for visualizing the differential abundance landscape of microbial species across population pairs. By faceting volcano plots by comparison, the figure allows simultaneous inspection of both the magnitude and statistical significance of microbial shifts between tribal communities. Highlighting species with |log<sub>2</sub>FC| > 2 and padj < 0.05 offers a stringent threshold to focus on biologically meaningful changes, supporting the identification of population-specific microbial signatures. This visual approach complements statistical summaries and highlights the comparative diversity and distinctness of gut microbial communities shaped by lifestyle, and ecology.

**Supplementary Figure 10: Population-specific and integrated microbial co-occurrence network centrality analysis showing the top 15 taxa ranked by degree, betweenness, and closeness centrality. Disease-associated taxa are highlighted in red.**

| All Community | Degree | Betweenness | Closeness | Positive_edges | Negative_edges | Total_edges | Role | Chenchu | Degree | Betweenness | Closeness | Positive_edges | Negative_edges | Total_edges | Role |
| --- | --- | --- | --- | --- | --- | --- | --- | --- | --- | --- | --- | --- | --- | --- | --- |
| Bacteroides_uniformis | 39 | 0.0714 | 0.5103 | 22 | 17 | 39 | Dual | Flavonifractor_plautii | 22 | 0.1212 | 0.4815 | 11 | 11 | 22 | Dual |
| Bacteroides_thetaiotaomicron | 36 | 0.0657 | 0.5077 | 20 | 16 | 36 | Dual | Phocaeicola_vulgatus | 22 | 0.0684 | 0.4764 | 11 | 11 | 22 | Dual |
| Bacteroides_ovatus | 34 | 0.0461 | 0.5051 | 18 | 16 | 34 | Dual | Bacteroides_ovatus | 22 | 0.0575 | 0.4643 | 12 | 10 | 22 | Dual |
| Parabacteroides_distasonis | 32 | 0.0920 | 0.5000 | 17 | 15 | 32 | Dual | Bacteroides_thetaiotaomicron | 21 | 0.0601 | 0.4573 | 11 | 10 | 21 | Dual |
| Flavonifractor_plautii | 28 | 0.0841 | 0.5156 | 14 | 14 | 28 | Dual | Bacteroides_caccae | 20 | 0.0684 | 0.4596 | 11 | 9 | 20 | Dual |
| Phocaeicola_vulgatus | 28 | 0.0592 | 0.5025 | 14 | 14 | 28 | Dual | Mogibacterium_kristiansenii | 17 | 0.0867 | 0.4136 | 7 | 10 | 17 | Dual |
| Odoribacter_splanchnicus | 27 | 0.0165 | 0.4420 | 16 | 11 | 27 | Dual | Parabacteroides_distasonis | 17 | 0.0624 | 0.4527 | 7 | 10 | 17 | Dual |
| Bacteroides_caccae | 26 | 0.0535 | 0.4853 | 13 | 13 | 26 | Dual | Bacteroides_uniformis | 17 | 0.0600 | 0.4174 | 10 | 7 | 17 | Dual |
| Megamonas_funiformis | 25 | 0.0885 | 0.4975 | 15 | 10 | 25 | Dual | Fusicatenibacter_saccharivorans | 16 | 0.0568 | 0.4417 | 11 | 5 | 16 | Dual |
| Bacteroides_stercoris | 25 | 0.0307 | 0.4783 | 17 | 8 | 25 | Dual | Megamonas_funiformis | 16 | 0.0414 | 0.4292 | 10 | 6 | 16 | Dual |
| Segatella_sinica | 24 | 0.0074 | 0.4541 | 9 | 15 | 24 | Dual | Segatella_sinensis | 16 | 0.0245 | 0.4354 | 6 | 10 | 16 | Dual |
| Prevotella_sp_Marseille_P4119 | 23 | 0.0042 | 0.4249 | 7 | 16 | 23 | Dual | Methanobrevibacter_smithii | 15 | 0.0607 | 0.4009 | 8 | 7 | 15 | Dual |
| Segatella_copri | 22 | 0.0442 | 0.4381 | 10 | 12 | 22 | Dual | Lacrimispora_amygdalina | 15 | 0.0439 | 0.4252 | 9 | 6 | 15 | Dual |
| Bacteroides_xylanisolvens | 22 | 0.0048 | 0.4439 | 13 | 9 | 22 | Dual | Oscillibacter_sp_ER4 | 15 | 0.0387 | 0.4194 | 6 | 9 | 15 | Dual |
| Parabacteroides_merdae | 21 | 0.0150 | 0.4583 | 12 | 9 | 21 | Dual | Gemmiger_formicilis | 14 | 0.0352 | 0.4194 | 10 | 4 | 14 | Dual |
| Irula | Degree | Betweenness | Closeness | Positive_edges | Negative_edges | Total_edges | Role | Jenu Kuruba | Degree | Betweenness | Closeness | Positive_edges | Negative_edges | Total_edges | Role |
| Clostridiaceae_bacterium_Marseille_Q4143 | 9 | 0.2281 | 0.3913 | 6 | 3 | 9 | Dual | Megamonas_funiformis | 9 | 0.2492 | 0.3382 | 7 | 2 | 9 | Dual |
| Oscillibacter_sp_ER4 | 9 | 0.1646 | 0.3629 | 5 | 4 | 9 | Dual | Oscillibacter_sp_MSJ_31 | 8 | 0.1862 | 0.2840 | 6 | 2 | 8 | Dual |
| Oscillibacter_valericigenes | 8 | 0.0920 | 0.3261 | 6 | 2 | 8 | Dual | Butyricicoccus_intestinisimiae | 6 | 0.0955 | 0.3046 | 2 | 4 | 6 | Dual |
| Bacteroides_uniformis | 8 | 0.0461 | 0.2922 | 6 | 2 | 8 | Dual | Blautia_faecis | 6 | 0.0629 | 0.2754 | 5 | 1 | 6 | Dual |
| Odoribacter_splanchnicus | 8 | 0.0280 | 0.2903 | 6 | 2 | 8 | Dual | Oscillibacter_valericigenes | 6 | 0.0521 | 0.2738 | 5 | 1 | 6 | Dual |
| Bacteroides_thetaiotaomicron | 7 | 0.1580 | 0.3435 | 5 | 2 | 7 | Dual | Bacteroides_uniformis | 6 | 0.0387 | 0.2722 | 5 | 1 | 6 | Dual |
| Parabacteroides_distasonis | 7 | 0.0295 | 0.2473 | 5 | 2 | 7 | Dual | Roseburia_faecis | 5 | 0.1691 | 0.2805 | 1 | 4 | 5 | Dual |
| Prevotella_sp_Marseille_P4119 | 6 | 0.0530 | 0.2980 | 1 | 5 | 6 | Dual | Parabacteroides_distasonis | 5 | 0.0558 | 0.3087 | 5 | 0 | 5 | Positive |
| Lachnospiraceae_bacterium | 6 | 0.0242 | 0.3000 | 4 | 2 | 6 | Dual | Parabacteroides_merdae | 5 | 0.0432 | 0.2788 | 4 | 1 | 5 | Dual |
| Bacteroides_ovatus | 6 | 0.0069 | 0.2866 | 5 | 1 | 6 | Dual | Phocaeicola_plebeius | 5 | 0.0135 | 0.2822 | 4 | 1 | 5 | Dual |
| Fusicatenibacter_saccharivorans | 5 | 0.0959 | 0.3103 | 4 | 1 | 5 | Dual | Candidatus_Cibionibacter_quicibialis | 4 | 0.2069 | 0.3087 | 2 | 2 | 4 | Dual |
| Lachnospiraceae_bacterium_AM48_27BH | 5 | 0.0938 | 0.3719 | 3 | 2 | 5 | Dual | Flavonifractor_plautii | 4 | 0.1878 | 0.3286 | 3 | 1 | 4 | Dual |
| Faecalibacterium_prausnitzii | 5 | 0.0705 | 0.2830 | 2 | 3 | 5 | Dual | Faecalibacterium_sp_HTFF | 4 | 0.1146 | 0.2949 | 2 | 2 | 4 | Dual |
| Blautia_faecis | 5 | 0.0683 | 0.3147 | 5 | 0 | 5 | Positive | Segatella_copri | 4 | 0.1011 | 0.2289 | 3 | 1 | 4 | Dual |
| Clostridium_fessum | 5 | 0.0417 | 0.3629 | 4 | 1 | 5 | Dual | Lacrimispora_amygdalina | 4 | 0.0391 | 0.2614 | 3 | 1 | 4 | Dual |
| Konda Savara | Degree | Betweenness | Closeness | Positive_edges | Negative_edges | Total_edges | Role | Kurumba | Degree | Betweenness | Closeness | Positive_edges | Negative_edges | Total_edges | Role |
| Bacteroides_uniformis | 26 | 0.1326 | 0.3973 | 15 | 11 | 26 | Dual | Bacteroides_caccae | 7 | 0.0374 | 0.3676 | 4 | 3 | 7 | Dual |
| Phocaeicola_vulgatus | 21 | 0.0531 | 0.3836 | 12 | 9 | 21 | Dual | Parabacteroides_distasonis | 6 | 0.0940 | 0.4386 | 5 | 1 | 6 | Dual |
| Bacteroides_caccae | 20 | 0.0306 | 0.3410 | 10 | 10 | 20 | Dual | Catenibacterium_tridentinum | 6 | 0.0520 | 0.3623 | 2 | 4 | 6 | Dual |
| Bacteroides_thetaiotaomicron | 18 | 0.0346 | 0.3410 | 12 | 6 | 18 | Dual | Coprococcus_comes | 5 | 0.0743 | 0.3289 | 4 | 1 | 5 | Dual |
| Bacteroides_ovatus | 18 | 0.0161 | 0.3755 | 9 | 9 | 18 | Dual | Bacteroides_uniformis | 5 | 0.0656 | 0.3846 | 4 | 1 | 5 | Dual |
| Segatella_sinensis | 17 | 0.0873 | 0.3693 | 8 | 9 | 17 | Dual | Odoribacter_splanchnicus | 4 | 0.0922 | 0.4098 | 2 | 2 | 4 | Dual |
| Flavonifractor_plautii | 17 | 0.0496 | 0.3371 | 9 | 8 | 17 | Dual | Dorea_longicatena | 4 | 0.0774 | 0.3623 | 3 | 1 | 4 | Dual |
| Odoribacter_splanchnicus | 16 | 0.2375 | 0.4009 | 11 | 5 | 16 | Dual | Alistipes_shahii | 4 | 0.0271 | 0.3846 | 2 | 2 | 4 | Dual |
| Bacteroides_xylanisolvens | 16 | 0.0514 | 0.3708 | 13 | 3 | 16 | Dual | Bacteroides_thetaiotaomicron | 4 | 0.0094 | 0.3205 | 4 | 0 | 4 | Positive |
| Parabacteroides_distasonis | 16 | 0.0428 | 0.3739 | 11 | 5 | 16 | Dual | Bacteroides_ovatus | 4 | 0.0035 | 0.3012 | 2 | 2 | 4 | Dual |
| Segatella_copri | 16 | 0.0339 | 0.3213 | 6 | 10 | 16 | Dual | Phocaeicola_vulgatus | 3 | 0.0484 | 0.3906 | 2 | 1 | 3 | Dual |
| Butyricicoccus_intestinisimiae | 15 | 0.0416 | 0.3589 | 6 | 9 | 15 | Dual | Blautia_wexlerae | 3 | 0.0222 | 0.2941 | 3 | 0 | 3 | Positive |
| Prevotella_SGB1675 | 15 | 0.0309 | 0.3589 | 5 | 10 | 15 | Dual | Prevotella_stercorea | 3 | 0.0040 | 0.3165 | 0 | 3 | 3 | Negative |
| Bacteroides_stercoris | 15 | 0.0301 | 0.3560 | 9 | 6 | 15 | Dual | Lachnospiraceae_bacterium | 2 | 0.0410 | 0.3472 | 1 | 1 | 2 | Dual |
| Mogibacterium_kristiansenii | 15 | 0.0258 | 0.3309 | 4 | 11 | 15 | Dual | Flavonifractor_plautii | 2 | 0.0222 | 0.2874 | 2 | 0 | 2 | Positive |

* Degree, betweenness, closeness, interaction type (positive, negative, or dual), and network association statistics for the top 15 microbial taxa identified in the integrated and population-specific microbial co-occurrence networks.

The network centrality analysis identified distinct yet partially conserved sets of highly connected taxa across the five tribal communities and the integrated microbial network. Taxa were ranked according to degree, betweenness, and closeness centrality, thereby identifying the fifteen most structurally central members of each network. In the integrated network, *Bacteroides uniformis* was the highest-ranked taxon, exhibiting the greatest degree (39), together with high betweenness (0.074) and closeness (0.502), indicating extensive connectivity throughout the network. This was followed by *Bacteroides thetaiotaomicron* (degree = 36, betweenness = 0.063, closeness = 0.494), *Bacteroides ovatus* (degree = 34, betweenness = 0.058, closeness = 0.490), and *Parabacteroides distasonis* (degree = 32, betweenness = 0.081, closeness = 0.485). Additional highly ranked taxa included *Flavonifractor plautii* (degree = 28), *Phocaeicola vulgatus* (degree = 28), *Odoribacter splanchnicus* (degree = 27), *Bacteroides caccae* (degree = 26), *Megamonas funiformis* (degree = 25), *Bacteroides stercoris* (degree = 25), *Segatella sinica* (degree = 24), *Prevotella* sp. Marseille-P4119 (degree = 23), *Segatella copri* (degree = 22), *Bacteroides xylanisolvens* (degree = 22), and *Parabacteroides merdae* (degree = 21). All fifteen taxa in the integrated network displayed dual interaction profiles, indicating the presence of both positive and negative significant associations. Comparison of the population-specific networks demonstrated both conserved and population-dependent patterns of structural organization. Two taxa, *Bacteroides uniformis* and *Parabacteroides distasonis*, were consistently identified among the top fifteen taxa in all five tribal communities, indicating their widespread structural importance across geographically distinct populations. *Bacteroides thetaiotaomicron* and *Bacteroides ovatus* were retained among the top-ranked taxa in four communities (Kurumba, Konda Savara, Irula, and Chenchu), whereas *Flavonifractor plautii*, *Bacteroides caccae*, and *Odoribacter splanchnicus* were shared by three communities. Several additional taxa, including *Phocaeicola vulgatus*, *Megamonas funiformis*, *Segatella sinensis*, *Segatella copri*, *Butyricicoccus intestinisimiae*, *Blautia faecis*, *Mogibacterium kristiansenii*, *Fusicatenibacter saccharivorans*, and *Oscillibacter* sp. ER4, occurred in two communities, whereas the remaining taxa were unique to individual populations, reflecting considerable population-specific variation in network topology. The Chenchu network was characterized by relatively high connectivity among *Phocaeicola vulgatus*, *Bacteroides ovatus*, *Bacteroides thetaiotaomicron*, *Bacteroides caccae*, *Bacteroides uniformis*, and *Parabacteroides distasonis*, each exhibiting degree values ranging from 15 to 20 and closeness values approaching 0.45–0.50. *Flavonifractor plautii* ranked among the fifteen most central taxa and exhibited the highest betweenness centrality within this community (approximately 0.17), while maintaining comparatively high degree and closeness values. This indicates that *Flavonifractor plautii* occupied one of the most topologically central positions within the Chenchu microbial network. The Irula network displayed a markedly different composition from the remaining populations and was dominated by *Clostridiaceae bacterium Marseille-Q4143*, *Oscillibacter* sp. ER4, *Oscillibacter valericigenes*, *Bacteroides uniformis*, *Bacteroides thetaiotaomicron*, *Odoribacter splanchnicus*, and *Parabacteroides distasonis*. Degree values ranged from approximately six to eight, while closeness values varied between approximately 0.32 and 0.43. Notably, none of the disease-associated taxa were retained among the fifteen highest-ranked taxa in the Irula network, indicating that the principal structural nodes of this community were exclusively composed of taxa not previously associated with multiple disease conditions.

The Jenu Kuruba network was primarily organized around *Megamonas funiformis*, *Oscillibacter* sp. MSJ-31, *Butyricicoccus intestinisimiae*, *Bacteroides uniformis*, *Parabacteroides distasonis*, *Parabacteroides merdae*, *Phocaeicola plebeius*, and *Roseburia faecis*. Degree values ranged from approximately five to eight, while closeness values remained between approximately 0.28 and 0.32. Among the disease-associated taxa, *Flavonifractor plautii* ranked within the top fifteen taxa and exhibited one of the highest betweenness values in the network (approximately 0.21), despite having only moderate degree and closeness values, indicating a comparatively prominent position within the network topology. The Konda Savara network exhibited the highest degree values among the population-specific analyses, with *Bacteroides uniformis* showing the greatest connectivity (degree = 24), followed by *Phocaeicola vulgatus*, *Bacteroides caccae*, *Bacteroides thetaiotaomicron*, *Bacteroides ovatus*, and *Parabacteroides distasonis*, all possessing degree values exceeding 20 and closeness values approaching 0.40. *Odoribacter splanchnicus* displayed the highest betweenness centrality within this community (approximately 0.23), indicating a comparatively prominent bridging position. Among the disease-associated taxa, *Flavonifractor plautii* remained within the top fifteen taxa, exhibiting intermediate degree, betweenness, and closeness values relative to the dominant *Bacteroides* taxa. The Kurumba network displayed comparatively lower overall connectivity, with maximum degree values of approximately seven. *Bacteroides caccae*, *Parabacteroides distasonis*, *Coprococcus comes*, *Bacteroides uniformis*, *Bacteroides ovatus*, *Bacteroides thetaiotaomicron*, and *Phocaeicola vulgatus* constituted the highest-ranked taxa. Two disease-associated taxa were identified among the top fifteen nodes. *Flavonifractor plautii* exhibited one of the highest betweenness values in the network (approximately 0.07), whereas *Blautia wexlerae* also ranked among the top fifteen taxa, although its degree, betweenness, and closeness values were comparatively lower than those of the dominant *Bacteroides* species. Notably, *Blautia wexlerae* was detected only in the Kurumba network, indicating a population-specific pattern of structural prominence. Among the six taxa previously identified as being associated with multiple disease conditions (*Bifidobacterium longum*, *Blautia wexlerae*, *Bacteroides fragilis*, *Ruminococcus gnavus*, *Collinsella aerofaciens*, and *Flavonifractor plautii*), only *Flavonifractor plautii* and *Blautia wexlerae* were represented among the highest-ranked taxa in the revised network analysis. *Flavonifractor plautii* showed the broadest distribution, occurring within the integrated network and in four of the five tribal populations (Chenchu, Jenu Kuruba, Konda Savara, and Kurumba), whereas it was absent from the Irula top-ranked network. In contrast, *Blautia wexlerae* was identified exclusively in the Kurumba community and did not rank among the highly central taxa in any other population or in the integrated analysis. The remaining disease-associated taxa (*Bifidobacterium longum*, *Bacteroides fragilis*, *Ruminococcus gnavus*, and *Collinsella aerofaciens*) were not retained among the top fifteen taxa in either the integrated or any population-specific network. These observations demonstrate that disease association and network centrality are not necessarily concordant and that only a subset of disease-associated taxa occupied structurally prominent positions under the statistically filtered co-occurrence network. Meanwhile, the recurrent identification of *Bacteroides uniformis*, *Parabacteroides distasonis*, *Bacteroides thetaiotaomicron*, and *Bacteroides ovatus* across multiple communities indicates that these taxa consistently represent the principal structurally central members of the healthy tribal gut microbial networks analyzed in this study.

**Supplementary Figure 11: Comparative relative abundance of six disease-associated bacterial taxa across five tribal populations.**

Kruskal-Wallis test results and pairwise (Wilcoxon rank sum test) comparisons indicate significant inter-population variation for *Bifidobacterium longum*, *Flavonifractor plautii*, and *Collinsella aerofaciens*, suggesting population-level microbial signatures with potential implications in inflammatory or metabolic conditions. Error bars represent standard deviation; *p < 0.05, **p < 0.01.

The analysis displays the relative abundance variation of six microbial taxa across populations using Kruskal-Wallis tests followed by pairwise Wilcoxon rank-sum comparisons. For *Bifidobacterium longum*, a statistically significant difference is observed across the populations (Kruskal–Wallis, p = 0.03), with the Chenchu population exhibiting the highest mean abundance and significantly differing from the Irula group. Additionally, a significant difference is also found between Irula and Konda Savara, indicating intra-population variation. *Blautia wexlerae* does not show an overall statistically significant difference across groups (p = 0.12), yet a notable pairwise divergence is evident between Chenchu and Konda Savara, suggesting localized shifts in this taxa's abundance despite non-significance at the global test level. *Bacteroides fragilis* shows higher mean abundance in the Chenchu group but lacks statistically significant differences among populations (p = 0.2), indicating moderate variability without strong intergroup separation. *Ruminococcus gnavus* presents a similar pattern with high variability in Chenchu but no significant overall or pairwise differences (p = 0.83), reflecting dispersed distribution without structured population-level clustering. *Flavonifractor plautii* reveals significant differences across populations (p = 0.0075), with elevated abundance in Chenchu and Jenu Kuruba. Several significant pairwise differences are found, including Chenchu vs Kurumba, Jenu Kuruba vs Konda Savara, and Jenu Kuruba vs Kurumba, suggesting this taxon is a strong differentiator among these groups. *Collinsella aerofaciens* does not exhibit a statistically significant global difference (p = 0.19), though a pairwise significance is noted between Kurumba and Irula, hinting at some community-specific elevation particularly in Kurumba. Collectively, the plot indicates that certain taxa such as *Bifidobacterium longum* and *Flavonifractor plautii* demonstrate population-specific abundance patterns, highlighting microbial signatures unique to particular tribal groups.

**Supplementary Figure 12: Population-stratified mean relative abundances of six disease-associated microbial taxa across lifestyle-associated variables, including acculturation level, chewing habit, sex, alcohol consumption, and smoking status.**

The figure reveals *Bifidobacterium longum* exhibits consistently higher mean abundance in more acculturated individuals (0.3776) compared to those with lower acculturation (0.2418), as well as in non-chewers (0.3423) versus chewers (0.1569), males (0.3569) versus females (0.2559), alcohol consumers (0.3334) versus non-drinkers (0.2952), and non-smokers (0.3281) over smokers (0.1997), suggesting a potentially protective profile influenced by specific lifestyle variables. *Blautia wexlerae* follows a broadly similar trend with increased abundance in more acculturated individuals (1.0045), males (1.0303), alcohol consumers (1.0087), and chewers (1.2151). However, its relative abundance is slightly lower in smokers (0.9414) compared to non-smokers (0.9962), indicating a more nuanced association with smoking. Notably, *Bacteroides fragilis*, *Ruminococcus gnavus*, and *Flavonifractor plautii* demonstrate pronounced enrichment in individuals with modernized or high-risk lifestyle features, including higher acculturation, male sex, alcohol consumption, and smoking. For instance, *Bacteroides fragilis* shows markedly higher levels in smokers (0.6735) compared to non-smokers (0.1944), as well as in alcohol users (0.4268) relative to abstainers (0.1627). Similarly, *Ruminococcus gnavus* is substantially enriched in smokers (0.8395), alcohol consumers (0.3597), and males (0.2900), suggesting strong associations with pro-inflammatory lifestyle factors. *Flavonifractor plautii* also shows elevated abundance in smokers (0.1052) and alcohol drinkers (0.0747), and appears more prevalent in males and acculturated individuals. In contrast, *Collinsella aerofaciens* displays a somewhat inverse association, with slightly higher abundance in less acculturated individuals (0.1284), chewers (0.1675), non-drinkers (0.1330), and non-smokers (0.1263), suggesting a distinct ecological response or potential resilience to modern lifestyle-related perturbations. Collectively, the observed patterns underscore a trend of microbiota compositional shifts aligned with lifestyle gradients, particularly highlighting the enrichment of specific pathobionts in association with acculturative and behavioral factors that may predispose individuals to gut dysbiosis and chronic disease risk.

**Supplementary Figure 13: Population-specific distribution of disease-associated candidate keystone taxa depicting their association with community structure (R²) and prevalence.**

The figure presents population-specific disease-associated candidate keystone taxa identified across five tribal communities, highlighting both their association with community structure (R²) and prevalence. *Bifidobacterium longum* emerged as a core taxon in both Chenchu and Irula groups, with high prevalence (0.71 and 0.97 respectively) and significant influence, particularly among the Chenchu (R² = 0.16, p = 0.001). *Ruminococcus gnavus* was also prevalent and influential in both Chenchu (R² = 0.13, prevalence = 0.76) and Jenu Kuruba (R² = 0.22, prevalence = 0.70), suggesting its role as a stable yet dynamic community structuring taxon across different lifestyles. *Flavonifractor plautii* showed strong population-level influence in both Chenchu (R² = 0.15) and Jenu Kuruba (R² = 0.22), though with only moderate prevalence, implying a conditional candidate keystone role potentially shaped by dietary components or host environment. *Blautia wexlerae*, found exclusively in the Konda Savara population, was universally present (prevalence = 1.00) and moderately influential (R² = 0.10), indicating its possible function as a dominant core species unique to this group. *Collinsella aerofaciens*, also in Konda Savara, exhibited high prevalence (0.82) but low influence (R² = 0.019), suggesting its presence as a consistent background taxon rather than a community driver. *Bacteroides fragilis* was moderately prevalent and significantly associated with three groups, Chenchu, Kurumba, and Konda Savara—with R² values ranging from 0.05 to 0.12, reinforcing its role as a widespread but context-dependent candidate keystone taxon. Collectively, the disease-associated taxa listed reflect how population-specific lifestyle factors likely shape gut microbial structure, with both highly prevalent and moderately prevalent taxa contributing to microbiome structuring in distinct yet overlapping ways. To understand the specific role of lifestyle-associated habits and practices, we conducted population-specific significance associations with the identified ‘candidate keystone taxa’ (see main text).

**Supplementary Figure 14: Cumulative contribution plot of gut-associated 489 microbial taxa based on SIMPER analysis, illustrating the ranked dissimilarity contributions of 1,542 identified taxa across studied populations.**

In the present study, Total Sum Scaling (TSS) normalization was adopted to convert raw counts into relative abundances because the downstream analytical framework, including Bray–Curtis dissimilarity, PERMANOVA, dbRDA, CCA, SIMPER, LEfSe, and microbial community comparisons, is conventionally based on relative abundance data and has been extensively applied using TSS-normalized microbiome datasets. TSS therefore provides a straightforward and biologically interpretable normalization strategy that is compatible with these community-level analyses. Although compositional approaches such as centered log-ratio (CLR) transformation are increasingly recommended for certain statistical applications, TSS was considered appropriate for the objectives and analytical framework employed here. Out of the 1,528 microbial taxa identified across all samples, a subset of 489 taxa was selected based on their cumulative contribution to intergroup compositional dissimilarity, as determined by Similarity Percentage (SIMPER) analysis. These 489 taxa collectively accounted for 91.13% of the total dissimilarity observed among the studied tribal populations, representing the most influential contributors to variation in gut microbial community structure. This cumulative threshold was strategically employed to retain taxa with substantial contributions to community structure while minimizing the influence of low-abundance or weakly contributing taxa that are unlikely to have a meaningful role in shaping community-level differences. Focusing on this refined subset enables the preservation of the principal patterns of community structure without overrepresenting rare or transient microbial signatures. Consequently, these taxa were considered representative of the gut microbiota in the context of the studied populations and were prioritized for downstream analyses, including the identification of candidate keystone taxa and community structure assessments. Given their consistent prevalence and strong contribution to inter-population microbiome divergence, this subset is herein referred to as the "gut-associated taxa." The use of a cumulative contribution-based selection approach enhances both the biological relevance and interpretability of downstream analytical outcomes by emphasizing taxa with established biological importance.

The selection strategy is further supported by the cumulative contribution plot, which visualizes the ranked dissimilarity contributions of individual taxa. The curve reveals a steep initial incline, indicative of a small number of taxa explaining the majority of compositional variation, followed by a long tail of taxa with minimal individual contributions. A distinct inflection point is observed near the 489th-ranked taxon, where the cumulative contribution reaches 91.13%. This visual pattern validates the choice of threshold by demonstrating that the selected subset captures nearly the entirety of the observed dissimilarity while efficiently excluding the remaining low-impact taxa.

To further dissect the distributional dynamics of taxon-level contributions, the 489 selected taxa were stratified into quartiles based on their ranked contribution values. Each quartile contained approximately 122–123 taxa. The distribution exhibited strong right-skewness, with the first quartile (Q1) alone accounting for 78.71% of the total cumulative dissimilarity. These taxa represent the dominant members driving microbiome variation across populations. The second quartile (Q2) contributed an additional 17.01%, cumulatively reaching 95.72%, indicating a secondary tier of biologically important but moderately contributing taxa. In contrast, the third (Q3) and fourth (Q4) quartiles contributed only 3.41% and 0.52%, respectively, highlighting their limited roles in shaping community-level differences.

This quartile-based dissection underscores the hierarchical nature of microbial contributions to intergroup dissimilarity. The disproportionate influence of the upper quartiles justifies their prioritization in functional and community-level analyses. Specifically, the taxa within Q1 and Q2, collectively explaining over 95% of community dissimilarity, are most appropriate for targeted exploration of candidate keystone taxa, community modelling, and co-occurrence network inference. These findings align with established concepts of species dominance and functional redundancy, supporting the notion that gut microbiome structure in these traditional populations is shaped by a relatively small set of consistently abundant and influential microbial taxa.

 **Supplementary Figure 15: Cross-cohort reproducibility of core candidate keystone taxa identified in the studied tribal populations using an independent, geographically distinct tribal validation cohort (Panel A), corresponding health-association profiles (Panel B), and cross-cohort concordance of stability scores between the Indian discovery and African validation cohorts (Panel C).**

The figure illustrates the cross-cohort reproducibility of core candidate keystone taxa originally identified in Indian tribal populations and their validation within an independent African tribal cohort. Of the 121 core candidate keystone taxa detected in the Indian tribes, 47 taxa were consistently observed in the African cohort, corresponding to a reproducibility rate of approximately 38.8%, indicating partial conservation of candidate keystone microbial signatures across geographically and culturally distinct traditional populations (Panel A). Among these reproducible taxa, 18 were further characterized as health-associated based on their positive and negative Health-Association Scores (HS), reflecting consistent associations with favourable host health states across cohorts (as related to the global health-association scores shown in the main text, Figures 7B and 7C). These taxa include well-established butyrate producers and metabolically relevant commensals such as *Fusicatenibacter saccharivorans*, *Ruminococcus bicirculans*, *Faecalibacterium prausnitzii*, *Roseburia intestinalis*, and *Anaerostipes hadrus*, which exhibited comparatively higher positive HS values, suggesting robust and reproducible health relevance. Lower, but still positive, HS values observed for additional taxa indicate moderate yet consistent health associations, whereas the presence of a single taxon with a negative score, *Blautia wexlerae*, highlights potential functional heterogeneity within the reproducible core as a disease-associated candidate keystone (Panel B). Panel C further evaluates the concordance of stability between the Indian discovery cohort and the independent African validation cohort. Stability scores were significantly correlated across cohorts (Spearman's ρ = 0.259, *P* = 0.00226), indicating overall conservation of candidate keystone stability despite substantial geographic separation. Taxa exhibiting the smallest differences in stability rankings, including *Phocaeicola vulgatus*, *Segatella brasiliensis*, *Parabacteroides merdae*, *Segatella copri*, *Alistipes shahii*, *Fusicatenibacter saccharivorans*, *Segatella sinensis*, *Segatella sinica*, and *Lachnospiraceae bacterium* AM48-27BH, demonstrated the greatest cross-cohort concordance. In contrast, *Mogibacterium kristiansenii, Eubacterium rectale, Parasutterella excrementihominis, Roseburia* sp. AM59_24XD, *Agathobaculum butyriciproducens, Faecalibacterium prausnitzii,* and *Faecalicatena amygdalina* exhibited the largest differences in stability rankings, suggesting greater cohort-specific variation in stability (Supplementary Table S11). Overall, the visualization captures both the degree of cross-population conservation of candidate keystone taxa and the spectrum of their health associations, supporting the existence of a conserved subset of gut microbial candidate keystone taxa with reproducible biological and health relevance across traditional human populations despite substantial geographic separation.

**Supplementary Table S13:** **Formal expression of candidate keystone taxa identification**

$$\begin{matrix} C_{\mathrm{core}} & =\{\text{ }t\in T:\text{ }R^{2}(t)\geq0.0199,\text{ }P_{\mathrm{envfit}}(t)<0.05,\text{ }P_{\mathrm{prev}}(t)\geq65\%\text{ }\}, \\ C_{\inf} & =\{\text{ }t\in T:\text{ }R^{2}(t)\geq0.0199,\text{ }P_{\mathrm{envfit}}(t)<0.05,\text{ }30\%\leq P_{\mathrm{prev}}(t)<65\%\text{ }\}, \\ C & =C_{\mathrm{core}}\cup C_{\inf}. \end{matrix}$$

| Symbol | Meaning |
| --- | --- |
| $\boldsymbol{T}$ | Set of all microbial taxa included in the analysis. |
| $\boldsymbol{t}$ | An individual microbial taxon, where $t\in T$. |
| $\boldsymbol{C}_{\text{core}}$ | Set of core-associated candidate keystone taxa, defined by significant community association ($R^{2}\geq0.0199$), significant permutation test ($P_{\text{envfit}}<0.05$), and prevalence ≥ 65%. |
| $\boldsymbol{C}_{\text{inf}}$ | Set of influential candidate keystone taxa, defined by significant community association ($R^{2}\geq0.0199$), significant permutation test ($P_{\text{envfit}}<0.05$), and prevalence between 30% and <65%. |
| $\boldsymbol{C}$ | Final set of candidate keystone taxa, obtained as the union of the core-associated and influential taxa ($C=C_{\text{core}}\cup C_{\text{inf}}$). |
| $\boldsymbol{R}^{\boldsymbol{2}}\boldsymbol{(t)}$ | Community association score of taxon $t$, estimated from the *envfit* analysis, representing the proportion of variation in microbial community ordination explained by the abundance of taxon $t$. |
| $\boldsymbol{P}_{\text{envfit}}\boldsymbol{(t)}$ | Permutation-derived significance value from the *envfit* analysis for taxon $t$. |
| $\boldsymbol{P}_{\text{prev}}\boldsymbol{(t)}$ | Prevalence of taxon $t$, expressed as the percentage of samples in which the taxon is detected. |
| $\boldsymbol{0.0199}$ | Empirically determined $R^{2}$threshold (65th percentile of the observed $R^{2}$distribution) used to identify taxa with relatively strong community associations. |
| $\boldsymbol{P}_{\text{envfit}}\boldsymbol{<0.05}$ | Criterion for statistically significant association between a taxon and microbial community structure. |
| $\boldsymbol{P}_{\text{prev}}\boldsymbol{\geq65}\boldsymbol{\%}$ | Threshold defining core-associated taxa, indicating widespread occurrence across samples. |
| $\boldsymbol{30}\boldsymbol{\%}\boldsymbol{\leq}\boldsymbol{P}_{\text{prev}}\boldsymbol{<65}\boldsymbol{\%}$ | Threshold defining influential taxa, indicating intermediate prevalence while retaining significant community association. |
| $\boldsymbol{\cup}$ | Union operator, indicating that the final candidate keystone set contains all taxa belonging to either $C_{\text{core}}$or $C_{\text{inf}}$. |
